# Tumor-Educated Platelet RNA for the Detection and (Pseudo)progression Monitoring of Glioblastoma

**DOI:** 10.1016/j.xcrm.2020.100101

**Published:** 2020-10-01

**Authors:** Nik Sol, Sjors G.J.G. in ‘t Veld, Adrienne Vancura, Maud Tjerkstra, Cyra Leurs, François Rustenburg, Pepijn Schellen, Heleen Verschueren, Edward Post, Kenn Zwaan, Jip Ramaker, Laurine E. Wedekind, Jihane Tannous, Bauke Ylstra, Joep Killestein, Farrah Mateen, Sander Idema, Philip C. de Witt Hamer, Anna C. Navis, William P.J. Leenders, Ann Hoeben, Bastiaan Moraal, David P. Noske, W. Peter Vandertop, R. Jonas A. Nilsson, Bakhos A. Tannous, Pieter Wesseling, Jaap C. Reijneveld, Myron G. Best, Thomas Wurdinger

**Affiliations:** 1 Brain Tumor Center Amsterdam, Cancer Center Amsterdam, Amsterdam UMC, VU University Medical Center, Amsterdam, the Netherlands; 2 Department of Neurology, Cancer Center Amsterdam, Amsterdam UMC, VU University Medical Center, Amsterdam, the Netherlands; 3 Department of Neurosurgery, Cancer Center Amsterdam, Amsterdam UMC, VU University Medical Center, Amsterdam, the Netherlands; 4 MS Center Amsterdam, Amsterdam UMC, VU University Medical Center, Amsterdam, the Netherlands; 5 Department of Neurology, Massachusetts General Hospital and Neuroscience Program, Harvard Medical School, Boston, MA, USA; 6 Department of Pathology, Cancer Center Amsterdam, Amsterdam UMC, VU University Medical Center, Amsterdam, the Netherlands; 7 Department of Pathology, Radboud University Medical Center, Nijmegen, the Netherlands; 8 Department of Biochemistry, Radboud Institute for Molecular Life Sciences, Nijmegen, the Netherlands; 9 Department of Medical Oncology, Maastricht Academical Medical Center, Maastricht, the Netherlands; 10 Department of Radiology and Nuclear Medicine, Amsterdam UMC, VU University Medical Center, Amsterdam, the Netherlands; 11 Department of Radiation Sciences, Oncology, Umeå University, Umeå, Sweden; 12 Princess Máxima Center for Pediatric Oncology, Utrecht, the Netherlands

**Keywords:** tumor-educated platelets, blood platelets, liquid biopsies, glioblastoma, machine learning, swarm intelligence

## Abstract

Tumor-educated platelets (TEPs) are potential biomarkers for cancer diagnostics. We employ TEP-derived RNA panels, determined by **swarm intelligence, to detect and monitor glioblastoma**. We assessed specificity by comparing the spliced RNA profile of TEPs from glioblastoma patients with multiple sclerosis and brain metastasis patients (validation series, n = 157; accuracy, 80%; AUC, 0.81 [95% CI, 0.74–0.89; p < 0.001]). Second, analysis of patients with glioblastoma versus asymptomatic healthy controls in an independent validation series (n = 347) provided a detection accuracy of 95% and AUC of 0.97 (95% CI, 0.95–0.99; p < 0.001). Finally, we developed the digitalSWARM algorithm to improve monitoring of glioblastoma progression and demonstrate that the TEP tumor scores of individual glioblastoma patients represent tumor behavior and could be used to distinguish false positive progression from true progression (validation series, n = 20; accuracy, 85%; AUC, 0.86 [95% CI, 0.70–1.00; p < 0.012]). In conclusion, TEPs have potential as a minimally invasive biosource for blood-based diagnostics and monitoring of glioblastoma patients.

## Highlights

TEP RNA enables blood-based brain tumor diagnostics
TEP RNA is dynamic throughout anti-tumor treatment
TEP RNA may be employed for therapy monitoring

## Graphical Abstract

**Figure:**
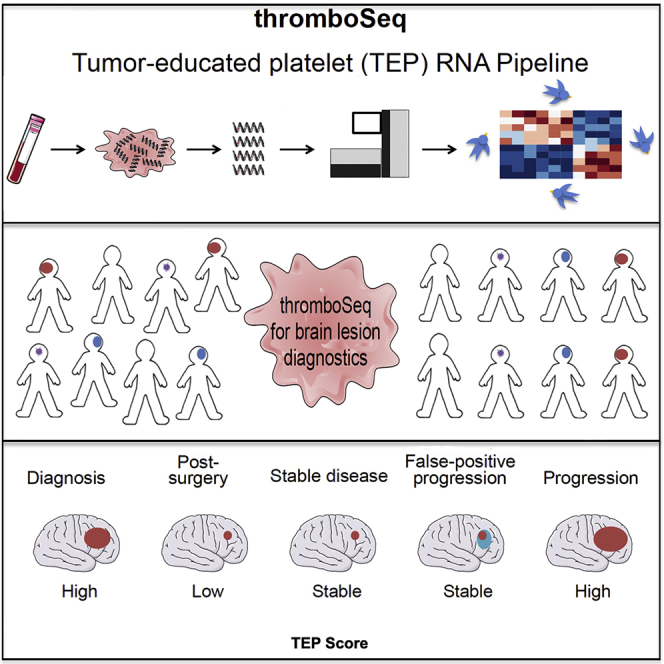

## Teaser

Sol et al. show that tumor-educated platelets (TEPs) can be employed for detection of glioblastoma. Glioblastoma TEP RNA profiles can be differentiated from patients with metastatic brain cancer or MS. Furthermore, the tumor signals in TEPs are dynamic, indicating that TEPs can be employed for blood-based therapy monitoring.

## Introduction

Several blood-based biosources, such as plasma, serum, plasma-derived extracellular vesicles, and circulating tumor cells, are currently being evaluated as liquid biopsies for many types of cancer.^1^^,^^2^ However, brain tumors are notoriously difficult to detect in blood.^3^, ^4^, ^5^, ^6^ Analysis of cerebrospinal fluid collected from patients with diffuse glioma revealed the presence of multiple molecular biomarkers.^7^ Alternatively, the presence of glioblastoma can only be identified in plasma DNA in less than 10% of patients while analyzing a plethora of point mutations and genomic rearrangements^8^ and in 60% of IDH1 mutant glioma patients when specifically analyzing *IDH1* mutants.^9^ In addition, circulating tumor cells were detected in 20%–73% of patients with glioblastoma, depending on the detection method applied.^10^, ^11^, ^12^ Detection of brain cancer in blood might leverage the use of such “liquid biopsies” for analysis of tumor progression, tumor recurrence, therapy response prediction, and monitoring^13^ and for differentiating glioblastoma tumor progression from false positive progression (pseudo-progression or radiation necrosis) after initial therapy.^3^

Blood platelets act as local and systemic responders during tumorigenesis and cancer metastasis^14^ and are exposed to tumor-induced platelet education, resulting in altered platelet behavior.^15^, ^16^, ^17^ We have shown that tumor-educated platelets (TEPs) are a potential biosource for blood-based cancer diagnostics^18^, ^19^, ^20^ by using the highly multiplexed biomarker detection platform thromboSeq.^21^ We previously detected glioblastoma with an accuracy of 84% in a 32-sample validation series among age-unmatched healthy (asymptomatic) controls.^18^ The TEP-derived RNA signatures allowed separation of patients with glioblastoma from those with metastases to the brain with 92% accuracy.^18^

In this study, we investigate the diagnostic power of thromboSeq for differentiation of glioblastoma from other brain lesions (i.e., brain metastases and active multiple sclerosis lesions), unifying unique TEP educational programs in neurological diseases. Furthermore, we show that TEP RNA profiles of patients with glioblastoma are different from those of asymptomatic healthy controls. Finally, we show that, in patients with glioblastoma, TEP RNA signatures correlated to tumor volume and tumor recurrence and may facilitate discrimination of true tumor progression from false positive progression.

## Results

### Unique Spliced RNA Profiles Can Be Identified in Platelets of Patients with Several Neurological Diseases

**Figure 1:**
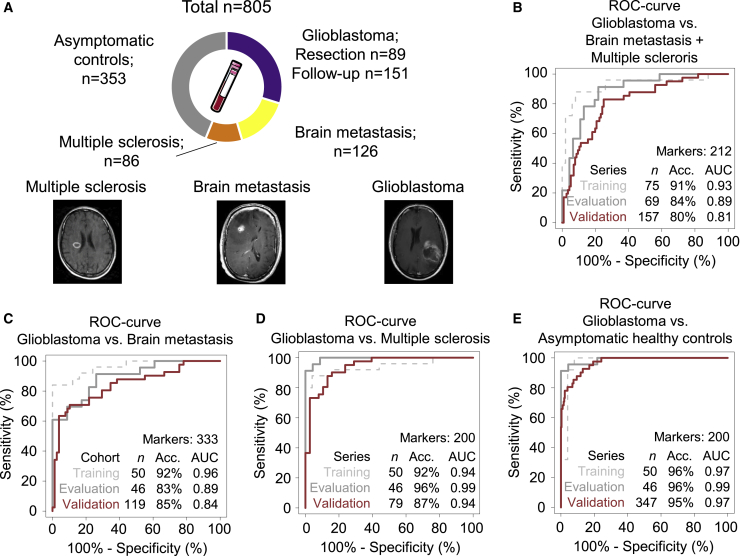
TEP RNA Profiling for Brain Tumor Diagnostics (A) Schematic overview of TEPs as biosource for liquid biopsies and number of groups and sample series sizes included. (B and C) receiver operating characteristics (ROC) curves of (B) “glioblastoma versus brain metastasis plus multiple sclerosis” algorithm, (C) “glioblastoma versus brain metastasis” algorithm, (D) “glioblastoma versus multiple sclerosis” algorithm, and (E) “glioblastoma versus asymptomatic healthy controls” algorithm, including the training series (dashed gray), evaluation series (gray), and validation series (red). Indicated are sample series sizes, best accuracy, and AUC value. Sample HC0068 and sample Maas-GBM-NICT-035G were inadvertently duplicated in the training or validation series because of a randomization code error identified after the validation process. See also Figure S1 and Tables S1 and S2A–S2E.

We first investigated whether several neurological diseases may differentially educate platelets, resulting in different spliced RNA profiles. For this, we prospectively collected and isolated platelet pellets from whole blood by differential centrifugation from 89 patients with primary glioblastoma collected on the day of first tumor resection (glioblastoma resection). From 52 of these 89 patients, follow-up blood samples were collected during postoperative chemo- and radiotherapy treatment (glioblastoma follow-up; 151 samples in total, 2–9 samples per patient). In addition, we collected blood from 126 patients with one or multiple brain metastases and 86 patients with relapsing-remitting multiple sclerosis (Figure 1A). Finally, we included platelet samples from 353 asymptomatic healthy controls without self-reported symptoms of neurological disorders or cancer, resulting in a total series size of 805 samples (Figure 1A; Table S1). Patients with brain metastases were diagnosed with different primary tumors: non-small cell lung carcinoma (NSCLC; n = 85), breast cancer (n = 15), melanoma (n = 15), renal cell cancer (n = 7), colorectal cancer (CRC; n = 1), esophagus cancer (n = 1), pancreatic cancer (n = 1), and an unknown primary tumor (n = 1). The majority of patients with brain metastases also had metastatic disease in other organs, including the liver, lungs, bones, and adrenal tissue (Table S1). Patients with multiple sclerosis were part of an early inception cohort in which patients were included at diagnosis and followed annually. These patients were diagnosed at least 10 years ago; all had clinically stable disease, and a subset of patients used disease-modifying drugs. Patient characteristics are provided in Table S1. From a small subset of 21 glioma patient samples, platelet counts were determined. No correlation was observed between platelet count and RNA concentration (Figure S1A). All samples were subjected to the thromboSeq platelet RNA sequencing pipeline (Figures S1B–S1D).^18^^,^^19^^,^^21^

To minimize potential confounding effect of previously identified confounding factors, we next selected, from this blood sample series, a sample set matched for age and whole-blood storage time, resulting in a matched series of 48 patients with glioblastoma at the time of first tumor resection, patients with brain metastases, patients with multiple sclerosis, and asymptomatic healthy controls. The median age was comparable among the four groups (Table 1), but it should be noted that the average age of patients with multiple sclerosis is lower because of a difference in time of disease onset compared with those with glioblastoma or brain metastases. Hence, we accepted the suboptimal results of age matching for patients with multiple sclerosis. We ensured that comparable numbers of samples were isolated within 24 or 48 h after blood withdrawal (Table 1), circumventing potential effects of blood storage time on the platelet-spliced RNA profiles.^19^

**Table 1:** Series Layout

Series	Group	n	Median age in years (IQR)	Blood storage time (<24 h)	Gender (% male; % unknown)
Training series	glioblastoma baseline	25	65 (23)	18 (72%)	64%; 0%
	multiple sclerosis	25	49 (17)	22 (88%)	24%; 12%
	brain metastasis	25	62 (11)	20 (80%)	40%; 0%
	asymptomatic controls	25	63 (30)	16 (64%)	44%; 0%
Evaluation series	glioblastoma baseline	23	63 (15)	18 (78%)	70%; 0%
	multiple sclerosis	23	47 (14.5)	19 (83%)	26%; 17%
	brain metastasis	23	65 (16)	18 (78%)	35%; 0%
	asymptomatic controls	23	63 (13.5)	16 (70%)	39%; 0%
Validation series	glioblastoma baseline	34	55 (18)	35 (85%)	68%; 15%
	multiple sclerosis	38	39 (43)	26 (68%)	18%; 32%
	brain metastasis	78	58 (14)	38 (49%)	49%; 0%
	asymptomatic controls	306	47 (29)	282 (92%)	33%; 8%

We first investigated the specificity of TEPs from glioblastoma patients by comparing the RNA profiles from patients with glioblastoma at the time of the first tumor resection with platelets from brain metastasis patients and patients with multiple sclerosis. For this, we randomly separated the total matched sample series into training, evaluation, and validation series (Table 1). The training samples were employed to select a spliced RNA biomarker panel and to build a machine learning support vector machine (SVM) algorithm, whereas the evaluation series was employed to further optimize the spliced RNA biomarker panel and performance of the SVM algorithm by swarm intelligence (STAR Methods). First, an algorithm biomarker panel of 212 platelet spliced RNAs was calculated to distinguish glioblastoma versus brain metastasis and multiple sclerosis. This resulted in a training series (n = 75) with an area under the curve (AUC) of 0.93 (95% confidence interval [CI], 0.85–1.00), an evaluation series (n = 69) with an AUC of 0.89 (95% CI, 0.81–0.97), and an independent validation series (n = 157) with an AUC of 0.81 (95% CI, 0.74–0.89; p < 0.001; Figure 1B; Table S2A). Second, another biomarker panel of 341 platelet-spliced RNAs was used in a multiclass comparison of glioblastoma versus multiple sclerosis versus brain metastasis. This resulted in a training series (n = 75) with an accuracy of 73%, an evaluation series (n = 69) with an accuracy of 77%, and a validation series (n = 157) with an accuracy of 75% (p < 0.001; Figure S1E; Table S2B). The patient groups were also analyzed separately and compared directly. For glioblastoma versus brain metastasis, a biomarker panel of 333 platelet-spliced RNAs was selected. This resulted in a training series (n = 50) with an AUC of 0.96 (95% CI, 0.92–1.00), an evaluation series (n = 46) with an AUC of 0.89 (95% CI, 0.81–0.98), and an independent validation series (n = 119) with an AUC of 0.84 (95% CI, 0.76–0.92; p < 0.001; Figure 1C; Table S2C). For glioblastoma versus multiple sclerosis, a biomarker panel of 200 platelet-spliced RNAs was selected. This resulted in a training series (n = 50) with an AUC of 0.94 (95% CI, 0.87–1.00), an evaluation series (n = 46) with an AUC of 0.99 (95% CI, 0.98–1.00), and an independent validation series (n = 79) with an AUC of 0.94 (95% CI, 0.89–0.99; p < 0.001; Figure 1D; Table S2D).

Because our data indicate that TEPs from glioblastoma patients have a unique spliced RNA profile, we next investigated whether patients with glioblastoma at the time of tumor resection can be differentiated from asymptomatic healthy controls. Following training and optimization of the thromboSeq classification algorithm with a biomarker panel of 200 platelet-spliced RNAs, a 347-sample validation series reached an accuracy of 68% for detection of glioblastoma at a specificity for detection of asymptomatic healthy controls of more than 98%. In this case, the training series (n = 50) resulted in an AUC of 0.97 (95% CI, 0.92–1.00), the evaluation series (n = 46) in an AUC of 0.99 (95% CI, 0.95–1.00), and the validation series (n = 347) in an AUC of 0.97 (95% CI, 0.95–0.99; p < 0.001; Figure 1E; Table S2E). We thus conclude that platelet-spliced RNA profiles may enable blood-based diagnosis of neuro-oncological and neuro-inflammatory conditions and are most likely not a response of aspecific platelet activation.

### TEP RNA Profiles of Patients with Glioblastoma Normalize toward Platelet RNA Profiles from Asymptomatic Healthy Controls following Tumor Resection

**Figure 2:**
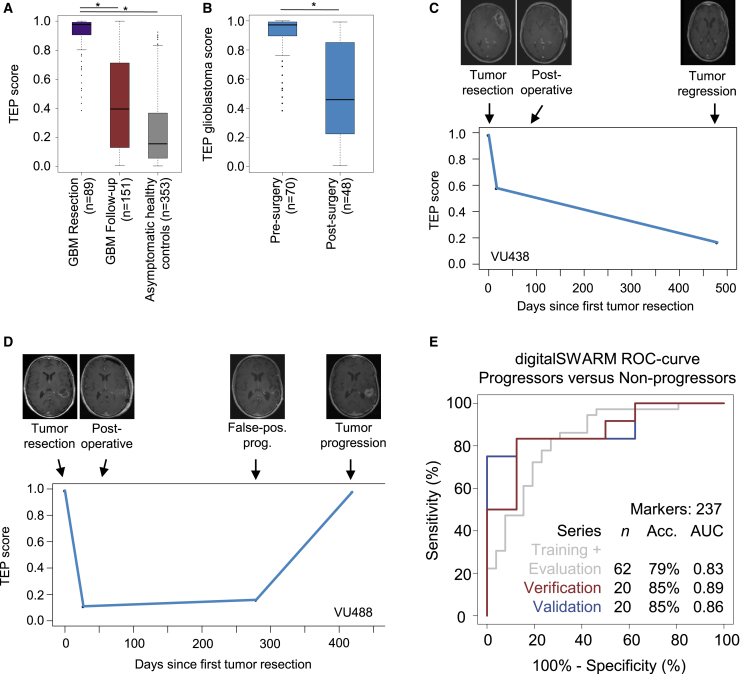
TEP RNA Signatures for Glioblastoma Therapy Monitoring and (False Positive) Progression Analysis (A) Boxplot of the TEP score (classification strength as output by the thromboSeq software) of glioblastoma at the moment of first tumor resection (n = 89), glioblastoma follow-up (n = 151), and asymptomatic healthy control (n = 353) samples classified in the “glioblastoma versus asymptomatic healthy controls” algorithm. Classification of glioblastoma follow-up samples (n = 151) results in significantly reduced TEP scores compared with glioblastoma samples collected at the moment of first tumor resection (n = 89). Per boxplot, the median, IQR, and 1.5 × IQR (whiskers) are shown. (B) Boxplot of the TEP score before and at the first time point after tumor resection (pre-surgery, n = 70; post-surgery, n = 48), indicating reduced TEP scores following (partial) tumor removal. Per boxplot, the median, IQR, and 1.5 × IQR (whiskers) are shown. (C and D) TEP score plotted during the therapy course, indicated as days since primary tumor resection, for patients VU438 (C) and VU488 (D), connected by a straight line. The MRI images acquired at each time point are shown at the top of the graph. Radiological evaluation of tumor growth is indicated below each MRI image. (E) ROC curve of the “progressive versus non-progressive” digitalSWARM classifier, including the combined training plus evaluation series (gray), verification series (red), and validation series (blue). Indicated are sample series sizes, best accuracy, and AUC value. See also Figure S2 and S3 and Table S2F.

Because we observed unique spliced RNA repertoires in TEPs of patients with glioblastoma at the time of tumor resection, we questioned whether these profiles might quantitatively decrease concordant with tumor volume. For this, following the blood sample at the time of tumor resection, we collected blood at several follow-up time points during the concurrent chemo-radiation phase together with magnetic resonance imaging (MRI) tumor visualization^22^ (52 unique patients, 151 platelet samples in total, 2–9 samples per patient; Table S1). Of one glioblastoma patient included in the follow-up sample collection, one pre-operative sample failed the sample processing quality checks (VU429 T0), but the follow-up samples were included in the analysis. Collection of follow-up samples was completed when there were no further treatment options or when a patient was lost to follow-up for any reason (STAR Methods). The sample collection schedule took into account the naturally occurring renewal of the platelet pool in blood following tumor resection because platelets have a 7- to 10-day lifespan.^23^ This follow-up sample series included glioblastoma patients with stable, progressive, and regressive disease as well as glioblastoma patients with false positive progression. We employed the “glioblastoma versus asymptomatic healthy controls” algorithm (Figure 1E) for classification of all glioblastoma baseline samples assigned to the validation series (n = 89) and glioblastoma follow-up samples (n = 151) and all asymptomatic healthy controls (n = 353). The “glioblastoma versus asymptomatic healthy controls” algorithm reports for each sample a binary classification result (“asymptomatic healthy control” or “glioblastoma”). However, in addition, the algorithm can also provide, for each sample, a quantitative score ranging from 0–1, representing the classification confidence score. The more pronounced the glioblastoma signal in a certain sample, the more this sample has a classification confidence score toward 1. We employed the classification confidence score to indicate the relative and quantitative signal of glioblastoma in platelet RNA profiles collected during therapy follow-up and called this classification confidence score the TEP score. First we observed that the TEP score of the blood samples collected from patients with glioblastoma just before tumor resection was significantly higher compared with those collected during the follow-up period (mean glioblastoma at tumor resection [n = 89], 0.91; mean glioblastoma follow-up [n = 151], 0.44; p < 2.2 × 10^−16^; independent Student’s t test; Figure 2A), and asymptomatic healthy controls (mean asymptomatic healthy controls [n = 353], 0.24; p < 2.2 × 10^−16^; independent Student’s t test; Figure 2A). To determine whether TEP-derived spliced RNA profiles do mirror the disease burden in the longitudinal sample collection, we analyzed the TEP score in 52 patients who were treated for their glioblastoma (Figures 2C and 2D; Data S1). When comparing pre-operative baseline blood samples with samples collected after tumor resection (median number of days after resection, 19; min-max, 10–33 days), we observed a mean decrease in TEP score after resection of 0.40 (p < 8.7e10^−11^, n = 70 versus n = 48 patients, unpaired independent Student’s t test; Figure 2B). These results indicate that the reduced tumor load after tumor resection significantly reduces the TEP score in blood. Moreover, we discovered a negative correlation between the TEP score calculated from the first postoperative sample and overall survival (r = −0.33, p = 0.03, Pearson’s correlation, n = 30 samples; Figure S2A). However, the contribution of potential additional prognostics factors, such as the age of the patient, Karnofsky performance score, and molecular tumor characteristics, cannot be excluded.

Subsequently, for each available time point, we determined the TEP score and matched the scores with the available MRI of the tumor (Figures 2C and 2D; Data S1). The patient with glioblastoma, VU438 (Figure 2C), had continuing lower volumes of gadolinium-enhancing tumor tissue in MRIs following tumor resection with a concomitant decrease in TEP score. Contrary, patient VU488, following a measure point with false positive progression, had true tumor progression with an increasing TEP score (Figure 2D). Interestingly, for some patients, the TEP score increased in blood prior to radiological tumor progression (Data S1), potentially indicating that a blood test may precede clinical and/or radiological tumor evolution. Although the correlation varied in individual cases, these results do indicate that the TEP score could possibly be employed for glioblastoma therapy monitoring.

False positive tumor progression remains a notorious challenge for therapy response monitoring using imaging modalities. To further investigate the potential value of the TEP-spliced RNA profiles following tumor resection and successful anti-tumor treatment, we questioned whether the TEP score would allow detection of false positive progression in glioblastoma treatment. Hence, we stratified all TEP samples from patients with glioblastoma collected during treatment follow-up into patients who had tumor progression at the time of blood collection (true progressive, n = 42), no tumor progression (stable disease or partial response [non-progressive], n = 40), or false positive progression (n = 20), scored according to the RANO criteria.^24^ False positive tumor progression was determined based on longitudinal clinical observation and combined-modality MRI, including perfusion MRI, but was not confirmed by tissue biopsy. We observed a decrease in average TEP score in patients in whom MRI enhancement was suspected for false positive progression compared with those with suspected true progressive tumors (mean true progressive [n = 42], 0.55; mean false positive progression [n = 20] 0.29; p = 0.001; independent Student’s t test; Figure S2B), and comparable with those with a non-progressive tumor (mean non-progressive [n = 40], 0.34; p = 0.003; Figure S2B). We next determined whether a dedicated classification algorithm capable of distinguishing progressors from non-progressors could be established; i.e., patients with stable disease, tumor regression, or false-positive progression. Unfortunately, for this particular analysis of tumor progression, the conventional particle swarm optimization (PSO)-enhanced thromboSeq classifier performed suboptimally, with an AUC of 0.71 (Figure S2C), in contrast to the other classifications showing AUCs of 0.81–0.97 for detection of glioblastoma (Figures 1B–1E). Hence, we decided to develop a variant of the classical PSO-enhanced thromboSeq SVM algorithm, which we called the digitalSWARM classification algorithm (STAR Methods). Briefly, this algorithm iteratively improves the biomarker RNA panel by binary including or excluding RNAs highly ranked according to ANOVA statistics and summarizes the analysis as a TEP score for tumor progression monitoring (Figure S3A). In addition, it randomly selects multiple training and evaluation series, enabling the algorithm to select the most optimal combination of training samples for independent verification and validation (Figure S3B). Of note, this algorithm resulted in similar outcomes for the glioblastoma diagnostics classifiers (Figure S3C). Next the digitalSWARM algorithm was used to determine a biomarker panel of 267 platelet RNAs, reaching an AUC of 0.86 in the validation series of the “progressors versus non-progressors” algorithm. In summary, the combined training and evaluation series (n = 62) reached an AUC of 0.83 (95% CI, 0.73–0.94), the verification series (n = 20) an AUC of 0.89 (95% CI, 0.73–1.00), and the validation series (n = 20) an AUC of 0.86 (95% CI, 0.70–1.00; p < 0.012; Figure 2E; Figure S3D; Table S2F). Importantly, the four patients with false positive progression included in the validation series were classified correctly as non-progressors. Gene Ontology analysis of the RNAs included in this spliced RNA biomarker panel enhanced in patients with tumor progression was associated with negative regulation of B cell proliferation and microglial cell activation (Figure S3E). Although Gene Ontology analyses should be interpreted with caution regarding platelet RNA profiles, as opposed to nucleated cells, these results potentially indicate that the platelet-immune axis is involved in glioblastoma tumor progression. Hence, we conclude that patients with false positive progression might be efficiently discriminated from those with true tumor progression by employing TEP RNA signatures, although large-scale external validation remains warranted.

## Discussion

Blood is a promising biosource to acquire molecular information regarding the presence and molecular make-up of a tumor.^1^ So far, it remains difficult to identify traces from brain tumors in blood.^8^ Our results show that patients with glioblastoma have markedly altered TEP-spliced RNA profiles that enable high-accuracy classification compared with TEP-spliced RNA profiles from asymptomatic healthy controls and patients with neuro-inflammatory or other (neuro)oncological conditions. This indicates that the measured platelet-spliced RNA profiles are not merely a result of aspecific platelet activation. We confirmed our previous observation^18^ that platelets collected from patients with brain metastases do contain differential spliced RNA profiles compared with those from patients with glioblastoma. Finally, we provide evidence that the glioblastoma fingerprint in TEPs may gradually regress following tumor resection but recurs during tumor progression, but the number of samples analyzed so far is limited.

Previously, it has been shown that increased pre-operative and post-chemo-radiotherapy platelet counts in patients with glioblastoma are associated with poorer survival.^25^, ^26^, ^27^ Also, glioblastoma cells induce platelet aggregation by expression of podoplanin, provoking venous thrombo-embolisms.^28^ Furthermore, platelets contain many growth factors that can be released upon activation, providing a pro-tumoral microenvironment.^14^^,^^29^^,^^30^ Hence, platelets may participate in progression of glioblastoma. Platelets are a potential anucleated source for RNA-based disease detection in blood.^18^, ^19^, ^20^^,^^31^, ^32^, ^33^, ^34^ They can be easily isolated in any clinical laboratory and contain high-quality RNA. Despite their anucleated state, platelets have the capability to inherit, splice, and translate their pre-mRNA content,^35^, ^36^, ^37^ but from the data collected in this study it cannot be deduced what effect the platelet RNA alterations may have on the platelets’ proteome.

In the presence of a tumor, platelets appear to be “educated” by the tumor environment. This may be achieved by (1) transcriptional alterations in the megakaryocyte; (2) differential splicing of platelet RNA upon external queues^36^, ^37^, ^38^, ^39^ derived from tumor, stromal, (peri)vascular, and/or immune cells; (3) sequestration of spliced (tumor-derived) RNAs;^20^^,^^31^ (4) platelet RNA alternative splicing events; or (5) the presence and evolution of differential platelet subpopulations in blood. Of particular interest here is tumor-bone marrow cross-talk, in which the glioblastoma tumor may influence bone marrow- or lung-resident megakaryocytes^40^ (for instance, via cytokines or extracellular vesicles), which may alter the transcriptional profile of megakaryocytes;^41^ however, direct biological evidence is lacking, as far as we are aware. In addition, megakaryocytes actively allocate RNA molecules into platelets,^35^ and therefore these megakaryocytes may actively influence the platelet RNA profiles. Because the platelet RNA content is tightly maintained with input from external events,^42^ local or systemic conditions might influence platelet RNA composition as well. Additional studies are required to determine which part of the observed platelet RNA profiles is subject to changes during thrombopoiesis and which part is acquired while in circulation.

Educated platelets may, in return, supply the tumor with pro-angiogenic factors^14^^,^^43^ or regulatory microRNA (miRNA) molecules.^44^ Because patients with a primary brain tumor could be differentiated from those with metastatic brain cancer, it may well be that the educational profiles are primarily derived from the primary non-central nervous system (CNS) tumor, but an effect of additional non-CNS metastases on the platelet-spliced RNA profiles cannot be excluded. Because we were able to distinguish patients with glioblastoma from those with the neuro-inflammatory condition multiple sclerosis, this dataset may indicate that the cancer signature is at least partly of tumorigenic origin and less likely due to peri-tumor inflammatory conditions only. Furthermore, platelets might be used to distinguish tumefactive MS from glioblastoma or primary CNS lymphoma. The gradual regression of the glioblastoma fingerprint in the platelet RNA samples collected following tumor resection and during the course of treatment supports this notion. It remains to be investigated why certain platelet RNA profiles from patients with glioblastoma included in the follow-up sample series show continuing TEP score reduction (Data S1), whereas their clinical course, supported by MRI, indicates tumor progression.

An advantage of the thromboSeq RNA sequencing (RNA-seq) platform is the multiplexed readout of ∼4,500 potential biomarkers profiled at once. The software tool enables selection of most contributing genes by employing swarm intelligence, leveraging the synergistic data in these RNA repertoires. The thromboSeq algorithm has shown high-accuracy performance with multiple tumor types,^18^^,^^19^ including neuro-oncological subtypes. It can be expected that, by including significantly more samples in the training process, the algorithms will be trained even more accurately, raising the overall classification performance. We provide evidence that the TEP-spliced RNA profiles are dynamic during tumor treatment. Glioblastomas cannot be fully eradicated; therefore, we cannot determine whether the platelet profile will completely normalize when the tumor is treated radically with chemoradiotherapy management and/or tumor resection. The exact “half-life” of the tumor signal in TEPs remains unknown and cannot be assessed in this tumor type. We observed a significant reduction in TEP score following tumor resection; on average, these samples were collected 19 (min-max, 10–33) days after surgery. This suggests that after the platelet pool is replaced, the TEP signal is also reduced. Like other liquid biopsies, these dynamic profiles possibly identify treatment failure before standard imaging techniques do.^45^, ^46^, ^47^ This potentially enables TEP RNA analysis to be used for therapy monitoring, perhaps also with other tumor types and therapy conditions.

Although the platelet pellets are isolated using a standardized protocol based on differential centrifugation, we cannot exclude that a part of the platelet RNA-derived signal is caused by co-isolation of other cell fragments of similar size. Contamination of remaining nucleated blood cells (e.g., leukocytes) in the platelet isolations cannot be ruled out and may at least partially influence the observed RNA profiles. Also, despite the fact that we aimed to minimize the effect of potential confounding factors by age and whole-blood storage time matching, residual (at present unknown) variables may still confound the identified classification accuracy. Follow-up studies should investigate the biological mechanism underlying the educational process of TEPs in patients with brain tumors. Also, the potential role of glioblastoma—platelet—megakaryocyte signaling should be investigated. As discussed previously, in patients with glioblastoma, platelet counts are associated with prognosis during adjuvant chemo-radiotherapy treatment.^48^ Also, the differential splicing patterns in platelets collected from patients with lower-grade glioma compared with those with glioblastoma and brain metastasis, is of interest, potentially providing blood-based opportunities to predict tumor progression of lower-grade toward higher-grade glioma. In addition, molecular tumor characteristics (e.g., IDH status, EGFR amplification, and other molecular characteristics) may be represented in TEP-spliced RNA profiles and deserve further investigation. Especially IDH status has shown effects on the coagulation status of glioma patients.^49^ Unfortunately, the number of samples with a known genetic tissue profile to classify patients into a glioma molecular subgroup were too low to draw any statistically valid conclusions, which is a limitation of this study, even though 4 patients with confirmed mutant IDH status were mostly classified correctly in the platelet-based predictions. Follow-up analysis should include an integrated morphological and molecular diagnosis.

Finally, integration of the TEP score together with MRI analysis may enhance detection and clinical management of patients with false positive glioblastoma progression. Prior to introduction of the TEP-based blood test in neuro-oncological clinics, thorough evaluation of pre-analytical variables and sample processing standardization as well as large-scale validation of TEP-spliced RNA profiles for brain tumor diagnostics and therapy monitoring are required.

### Limitations of Study

One limitations of this study is the low sample numbers, especially for detection of false positive progression; more samples would lead to a more robust prediction algorithm. Second, a head-to-head comparison with MRI modalities used to make false positive progression more or less likely is missing. Furthermore, more imaging and blood collection time points are necessary to determine whether treatment failure can be identified in TEPs before standard imaging techniques do. Another limitation is the unknown genetic profile of most GBM samples; especially IDH status could influence the RNA profile of platelets. Although several hypotheses of platelet education are discussed, the exact mechanism of platelet education remains unclear. All of these are important aspects that will need to be addressed in future studies.

## STAR★Methods

### Key Resources Table

**Table:** 

REAGENT or RESOURCE	SOURCE	IDENTIFIER
**Biological Samples**		

805 blood platelet samples	This study	Table S1

**Chemicals, Peptides, and Recombinant Proteins**		

RNALater solution	Thermo Scientific	Cat# AM7020

**Critical Commercial Assays**		

mirVana miRNA isolation kit	Ambion, Thermo Scientific	Cat# AM1560
SMARTer Ultra Low RNA Kit for Illumina Sequencing version 3	Clontech	Cat# 634853
Truseq Nano DNA Sample Prep Kit	Illumina	Cat# FC-121-4001
RNA picochip and reagents, Bioanalyzer 2100	Agilent	Cat# 5067-1513
DNA 7500 chip and reagents, Bioanalyzer 2100	Agilent	Cat# 5067-1506
DNA High Sensitivity chip and reagents, Bioanalyzer 2100	Agilent	Cat# 5067-4626

**Deposited Data**		

Raw and processed RNA-seq data	This study	GEO: 156902

**Software and Algorithms**		

Trimmomatic (version 0.22)	Bolger et al.^54^	http://www.usadellab.org/cms/?page=trimmomatic
STAR (version 2.3.0)	Dobin et al.^55^	https://github.com/alexdobin/STAR
HTSeq (version 0.6.1)	Anders et al.^56^	https://www-huber.embl.de/HTSeq/doc/overview.html
Picardtools (version 1.115)	Broad Institute, USA	https://broadinstitute.github.io/picard/
Samtools (version 1.115)		http://samtools.sourceforge.net
MATLAB (version R2015b)	The MathWorks Inc., USA	https://nl.mathworks.com/products/matlab.html
R (version 3.3.0)		https://www.r-project.org
R-studio (version 0.99.902)		https://rstudio.com/
Bioconductor package edgeR (version 3.12.1)		https://bioconductor.org/packages/release/bioc/html/edgeR.html
Bioconductor package EDASeq (version 2.4.1)		http://bioconductor.org/packages/release/bioc/html/EDASeq.html
Bioconductor package PPSO (version 0.9-9991)		https://github.com/TillF/ppso
Bioconductor package RUVSeq (version 1.4.0)		http://bioconductor.org/packages/release/bioc/html/RUVSeq.html
R-package e1071 (version 1.6-7)	CRAN	https://cran.r-project.org/web/packages/e1071/index.html
R-package Optunity (version 1.0)	STADIUS lab	https://optunity.readthedocs.io/en/latest/
R-package pROC (version 1.8)	CRAN	https://cran.r-project.org/web/packages/pROC/index.html
R-package ROCR (version 1.0-7)	CRAN	https://cran.r-project.org/web/packages/ROCR/index.html
ThromboSeq algorithm v1.4		https://github.com/MyronBest/thromboSeq_source_code

### Resource Availability

#### Lead Contact

Further information and requests for resources and reagents should be directed to and will be fulfilled by the Lead Contact Thomas Wurdinger (t.wurdinger@amsterdamumc.nl).

#### Materials Availability

This study did not generate new unique reagents.

#### Data and Code Availability

The raw sequencing data reported in this paper has been deposited into the NCBI GEO database under accession number 156902. All employed bioinformatics software and code can be found online (https://github.com/MyronBest; thromboSeq_source_code_v1.4 [to be released]).

### Experimental Model and Subject Details

#### Study design and sample selection

Peripheral whole blood was drawn by venipuncture from brain cancer patients, patients with multiple sclerosis, and asymptomatic healthy individuals at the various medical institutions in Europe and the USA. Whole blood was collected in 4-, 6-, or 10-mL EDTA-coated BD Vacutainers. Cancer patients were diagnosed by clinical, radiological and pathological examination, and were confirmed to have detectable tumor tissue load at the time of blood collection. Age- and whole blood storage time-matching was performed retrospectively, iteratively matching samples by excluding and including patients with glioblastoma at the time of first tumor resection, patients with brain metastasis, patients with multiple sclerosis, and asymptomatic healthy controls, aiming at a similar median age and age-range between groups. A detailed overview of the included samples, demographic characteristics, the hospital of origin, time between blood collection and platelet isolation (whole blood storage time), as well as an overview for which analyses and classifiers the samples were used is provided in Table S1. Asymptomatic healthy controls were at the moment of blood collection, or previously, not diagnosed with cancer, but were not subjected to additional tests confirming the absence of cancer. None of the patients with multiple sclerosis had a malignancy at the moment of blood collection. This study was conducted in accordance with the principles of the Declaration of Helsinki. Approval for this study was obtained from the institutional review board and the ethics committee at each participating hospital. Each participant signed informed consent. Clinical follow-up of asymptomatic healthy controls is not available due to anonymization of these samples according to the ethical rules of the hospitals.

#### Clinical data annotation

For collection and annotation of clinical data, patient records were manually queried for demographic and clinical variables, i.e., age, gender, type of tumor, metastases, details of current and prior treatments, survival rates, and co-morbidities. Blood samples from patients with glioblastoma were collected at the day of surgery (i.e., before surgery) and/or during treatment, respectively baseline and follow-up samples. During the follow-up period blood was preferably collected at the time of, or within weeks, of follow-up MRIs and always before a new treatment session. Sample collection was discontinued at the time of tumor progression, or because of any other reason through which a patient had become lost to follow-up (e.g., withdrawal of informed consent, discontinuation of treatment, continuation of treatment in another hospital, or death). Treatment response assessment of patients was done by MR-imaging and was performed by experienced neuroradiologists according to the updated RANO criteria, and scored as progressive disease (PD), stable disease (SD), partial response (PR), or complete response (CR).^50^, ^51^, ^52^ False-positive progression was determined by subsequent MR-imaging,^53^ including multiple MR modalities and perfusion MRI. A tissue biopsy is not required since tissue biopsies cover only a very limited area of the brain tumor and are therefore prone to sampling error. In combination with the additional surgical risk for the patient it is generally not used to confirm glioblastoma false-positive progression. All clinical data was anonymized and stored in a secured database.

### Method Details

#### Blood processing and platelet isolation

Whole blood samples in 4-, 6-, or 10-mL EDTA-coated BD Vacutainer tubes were processed using standardized protocols within 48 hours as described previously.^18^, ^19^, ^20^, ^21^ Whole blood collected at the Amsterdam UMC location VUmc, the Utrecht Medical Center, the Medical University of Vienna, the Radboud University Medical Center Nijmegen, the University Hospital of Maastricht, and the Netherlands Cancer Institute was subjected to platelet isolation within 12 hours after blood collection. Whole blood samples collected at Massachusetts General Hospital Boston and at the Amsterdam UMC location AMC were stored overnight and processed after 24 hours. Platelet pellets were isolated as described previously.^18^^,^^19^^,^^21^ To isolate platelets, platelet rich plasma (PRP) was separated from nucleated blood cells by a 20-minute 120xg centrifugation step, after which the platelets were pelleted by a 20-minute 360xg centrifugation step. In order to reduce the risk of leukocyte contamination pelleted in the buffy coat, removal of 9/10^th^ of the PRP has to be performed carefully. The remaining leukocyte yield with this isolation method is *∼*1 to 5 leukocytes per 1 million platelets.^18^ Centrifugations were performed at room temperature. Finally, platelet pellets were carefully resuspended in RNAlater (Life Technologies) and after overnight incubation at 4°C frozen at −80°C. Platelet pellets in RNAlater can be stored at least for up to five years at −80°C while maintaining high-quality.

#### Total RNA isolation, SMARTer amplification, and Truseq library preparation

Preparation of samples for sequencing was performed in batches as described previously,^18^^,^^19^^,^^21^ and included a mixture of clinical conditions per batch. For platelet RNA isolation, frozen platelets were thawed on ice and total RNA was isolated using the mirVana miRNA isolation kit (Ambion, Thermo Scientific, AM1560). Platelet RNA was eluted in 30 μL elution buffer. We evaluated the platelet RNA quality using the RNA 6000 Picochip (Bioanalyzer 2100, Agilent), and included, as a quality standard for subsequent experiments, only platelet RNA samples with a RIN-value > 7 and/or distinctive rRNA curves. All Bioanalyzer 2100 quality and quantity measures were collected from the automatically generated Bioanalyzer result reports using default settings, and after critical assessment of the reference ladder (quantity, appearance, and slope). High-quality samples were subjected to cDNA synthesis and amplification using the SMARTer Ultra Low RNA Kit for Illumina Sequencing v3 (Clontech, cat. nr. 634853). Prior to amplification, all samples were diluted to ∼500 pg/μL total RNA and again the quality was determined and quantified using the Bioanalyzer Picochip. For samples with a stock yield below 400 pg/μL, a volume of two or more microliters of total RNA (up to ∼500 pg total RNA) was used as input for the SMARTer amplification. Quality control of amplified cDNA was measured using the Bioanalyzer 2100 with DNA High Sensitivity chip (Agilent). All SMARTer cDNA synthesis and amplifications were performed together with a negative control, which was required to be negative by Bioanalyzer analysis. Samples with detectable fragments in the 300-7500 bp region were selected for further processing. For labeling of platelet cDNA for sequencing, all amplified platelet cDNA was first subjected to nucleic acid shearing by sonication (Covaris Inc) and subsequently labeled with single index barcodes for Illumina sequencing using the Truseq Nano DNA Sample Prep Kit (Illumina, cat nr. FC-121-4001). To account for the low platelet cDNA input concentration, all bead clean-up steps were performed using a 15-minute bead-cDNA binding step and a 10-cycle enrichment PCR. All other steps were according to manufacturer’s protocol. Labeled platelet DNA library quality and quantity was measured using the DNA 7500 chip or DNA High Sensitivity chip (Agilent). High-quality samples with product sizes between 300-500 bp were pooled (12-19 samples per pool) in equimolar concentrations for thromboSeq and submitted for 100 bp Single Read sequencing on the Illumina Hiseq 2500 platform using version 4 sequencing reagents.

### Quantification and Statistical Analysis

#### Processing of raw RNA-sequencing data

Raw RNA-seq data of platelets encoded in FASTQ-files were subjected to a standardized RNA-seq alignment pipeline, as described previously.^18^^,^^19^^,^^21^ In summary, RNA-seq reads were subjected to trimming and clipping of sequence adapters by Trimmomatic (v. 0.22),^54^ mapped to the human reference genome (hg19) using STAR (v. 2.3.0),^55^ and summarized using HTSeq (v. 0.6.1), which was guided by the Ensembl gene annotation version 75.^56^ All subsequent statistical and analytical analyses were performed in R (version 3.3.2) and R-studio (version 0.99.903). Of samples that yielded less than 0.2x10^6^ intron-spanning reads in total after sequencing, we again sequenced the original Truseq preparation of the sample and merged the read counts generated from the two individual FASTQ-files after HTSeq count summarization (performed for n = 31 samples). Genes encoded on the mitochondrial DNA and the Y chromosome were excluded from downstream analyses. Sample filtering was performed by assessing the library complexity, which is partially associated with the intron-spanning reads library size. First, we excluded the genes that yielded < 30 intron-spanning reads in > 90% of the cohort for all platelet samples that were sequenced. This resulted in this platelet RNA-seq library in 4,487 different genes detected with sufficient coverage. For each sample, we quantified the number of genes for which at least one intron-spanning read was mapped, and excluded samples with < 750 detected genes. Following, we performed a leave-one-sample-out cross-correlation analysis to exclude platelet samples that show low intersample correlation (Pearson’s correlation threshold: 0.4). Finally, we excluded platelet RNA samples with median logCPM < 3, resulting in a final data series of n = 805 samples (n = 353 asymptomatic healthy controls, n = 240 glioblastoma, n = 126 brain metastasis, and n = 86 multiple sclerosis; Figure 1A; Table S1). To prevent potential plasma DNA from contributing to our computational platelet RNA analyses, we only selected spliced intron-spanning RNA reads.

#### Support Vector Machine (SVM)-based algorithm development and particle swarm optimization (PSO)-driven SVM-parameter optimization

The PSO-enhanced algorithm was extensively described previously.^19^^,^^21^ Briefly, the algorithm employs a training and evaluation series for gene panel selection and algorithm development, of which selection parameters are optimized by PSO. For our classifiers we employed 100 particles with 6 iterations, and optimized four steps of the generic classification algorithms, i.e., (i) the iterative correction module threshold used for selection of genes identified as stable genes among the library size, (ii) the FDR-threshold included in the differential splicing filter applied to the results of the likelihood ANOVA test, (iii) the exclusion of highly correlated genes selected after the likelihood ANOVA test, and (iv) number of genes passing the recursive feature elimination (RFE)-algorithm. Predefined ranges were submitted to the PSO-algorithm for every classification task presented in this study. To identify a potential algorithm threshold at which the maximum number of patients with glioblastoma may be identified with a predefined specificity for asymptomatic healthy controls, we geared the algorithm readout in the evaluation series toward 95% specificity. The software code automatically applies the algorithm predictive strength threshold selected in the evaluation series to the validation series.

#### Performance measurement of the PSO-enhanced thromboSeq algorithm

All classification experiments were performed with the PSO-enhanced thromboSeq algorithm, using parameters optimized by PSO. All random selection procedures were performed using the *sample*-function as implemented in R. For assignment of samples per series to the training and evaluation series, only the number of samples per clinical group was balanced, whereas other potentially contributing variables were not stratified at this stage (assuming random distribution among the groups). Performance of the training series was assessed by a leave-one-out cross validation approach (LOOCV, see also Best et al.^18^). The list of stable genes among the initial training series, determined RUV-factors for removal, and final gene panel determined by swarm-optimization of the training-evaluation series were used as input for the LOOCV procedure. As a control for internal reproducibility, we randomly sampled training and evaluation series, while maintaining the validation series and the swarm-guided gene panel of the original classifier, and performed 1000 training and classification procedures. As a control for random classification, class labels of the samples used by the SVM-algorithm for training of the support vectors were randomly permutated (n = 1000 iterations), while maintaining the swarm-guided gene list of the original classifier. P values were calculated accordingly, as described previously.^18^ Results were presented in receiver operating characteristics (ROC)-curves, and summarized using area under the curve (AUC)-values, as determined by the ROCR-package in R. AUC 95% confidence intervals were calculated according to the method of Delonge using the *ci.auc*-function of the pROC-package in R.

#### digitalSWARM classification algorithm

DigitalSWARM in essence iteratively and digitally (binary) selects RNAs to be included in the biomarker panel. In addition, digitalSWARM enables for inclusion of a verification series that allows for selection of multiple and ultimately the best combination of samples assigned to the training and evaluation series, a potential pitfall of the conventional PSO-enhanced thromboSeq classifier.^21^ The algorithm performs the following steps of which the full dataset is employed as input data; first, random selection of *n* training and evaluation series (n = 200 by default), with only one locked evaluation and one locked validation series. Then, the algorithm analyzes a grid of multiple potential confounding factor (e.g., library size) thresholds for RUV-correction. For each setting an ANOVA-comparison employing the training series is performed and the potential confounding factor setting of which the lowest ANOVA false discovery rate could be achieved is selected as the most optimal RUV-correction threshold. Next, a preliminary biomarker RNA panel is selected (by default p value < 0.1, logCPM > 3, no expression of RNAs encoded on the Y chromosome) separately for RNAs with increased or decreased numbers of reads mapping to splice junctions. Following, for both biomarker RNA panels, a particle swarm optimization (PSO)-algorithm iteratively enables for in- or exclusion (‘1’ or ‘0’) of RNAs (default number of particles: 100, default number of iterations: 50). The performance of each biomarker RNA panel proposed by PSO is tested by calculating the median expression level of all samples in the evaluation series, progressive and non-progressive separately, and performing an independent Student’s t test comparison. The ultimately best biomarker spliced RNA panel, either with in- or decreased RNAs, is employed for calculating a companion TEP score. This score is calculated by the median log_2_ normalized counts of either biomarker RNA panel and for the progressive and non-progressive samples separately. For the training, evaluation, and verification series an area-under-the curve (AUC)-value is calculated, and for all iterations and each biomarker panel selected the best verification series AUC is selected. Employing this particular training and evaluation series and biomarker RNA panel the validation series is classified and summarized. 30% of the samples in the dataset were assigned to both the training and evaluation series, whereas 20% was assigned to both the verification and validation series. The specificity of the biomarker RNA panel is assessed by randomly shuffling the labels of the training and evaluation series samples before the procedure (n = 1000 times), thereby requiring at least 20% of the sample labels to be different as compared to the original sample label.

#### Gene ontology analysis

Gene ontology analysis was performed on the 5^th^ of September 2019 with the PantherDB-database, performing gene ontology analysis for biological processes (http://www.pantherdb.org). PANTHER overrepresentation test was selected, employing the *Homo sapiens* reference list (n = 20,996 genes), which were compared to RNAs with enhanced spliced junction reads in the ‘non-progressive’-group (n = 126) and RNA with enhanced spliced junction reads in the ‘progressive’-group (n = 131) in the ‘progressive versus non-progressive’-algorithm (Figure 2E). Fisher’s exact test was employed to calculate statistical significance and the top five hits sorted by enrichment score were plotted in barplots.

## Supplemental Information

Document S1. Figures S1–S3 and Data S1

Table S1. Patient Characteristics Detailed overview of all samples included in this study (n = 851), including patient characteristics, and inclusion in the multiple algorithms developed in this study. Indicated are sample names, patient group, classification group, hospital of blood collection, storage time between blood collection and platelet isolation (blood storage time), age in years, gender, smoking, metastasis, treatment, and response to treatment. In addition, for each sample is stated to which cohort (training, evaluation or validation) the sample is assigned in the different classifiers. In column “Hospital” VUMC is VU University Medical Center, Amsterdam, the Netherlands; NKI is Dutch Cancer Institute, Amsterdam, the Netherlands; AMC is Academical Medical Center, Amsterdam, the Netherlands; UMCU is Utrecht Medical Center, Utrecht, the Netherlands; MGH is Massachusetts General Hospital, Boston, USA; VIENNA is the Medical University of Vienna, Austria; RAD is the Radboud University Medical Center Nijmegen, the Netherlands; MAAS is the University Hospital of Maastricht, the Netherlands. Column “Gender”: M, male; F, female. Column “Smoking”: Y, current; N, never; F, former. Column “Metastasis”: Y, yes; N, no; NA = not applicable.

Table S2. Biomarker RNA Panels Biomarker RNA panels of the classification algorithms. Indicated for each biomarker panel are included the Ensembl IDs, HGNC symbols, and gene descriptions.

Document S2. Article plus Supplemental Information
