# Supplementary material for: Tumor-Educated Platelet RNA for the Detection and (Pseudo)progression Monitoring of Glioblastoma
Source: Cell Rep Med. 2020 Oct 1;1(7):100101. doi: 10.1016/j.xcrm.2020.100101 (PMC7576690; doi:10.1016/j.xcrm.2020.100101)
Supplement: Document S1. Figures S1–S3 and Data S1 [file mmc1.pdf]

Cell Reports Medicine, Volume 1

Supplemental Information

Tumor-Educated Platelet RNA for the Detection and (Pseudo)progression Monitoring of Glioblastoma

Nik Sol, Sjors G.J.G. in `t Veld, Adrienne Vancura, Maud Tjerkstra, Cyra Leurs, François Rustenburg, Pepijn Schellen, Heleen Verschueren, Edward Post, Kenn Zwaan, Jip Ramaker, Laurine E. Wedekind, Jihane Tannous, Bauke Ylstra, Joep Killestein, Farrah Mateen, Sander Idema, Philip C. de Witt Hamer, Anna C. Navis, William P.J. Leenders, Ann Hoeben, Bastiaan Moraal, David P. Noske, W. Peter Vandertop, R. Jonas A. Nilsson, Bakhos A. Tannous, Pieter Wesseling, Jaap C. Reijneveld, Myron G. Best, and Thomas Wurdinger

Figure S1

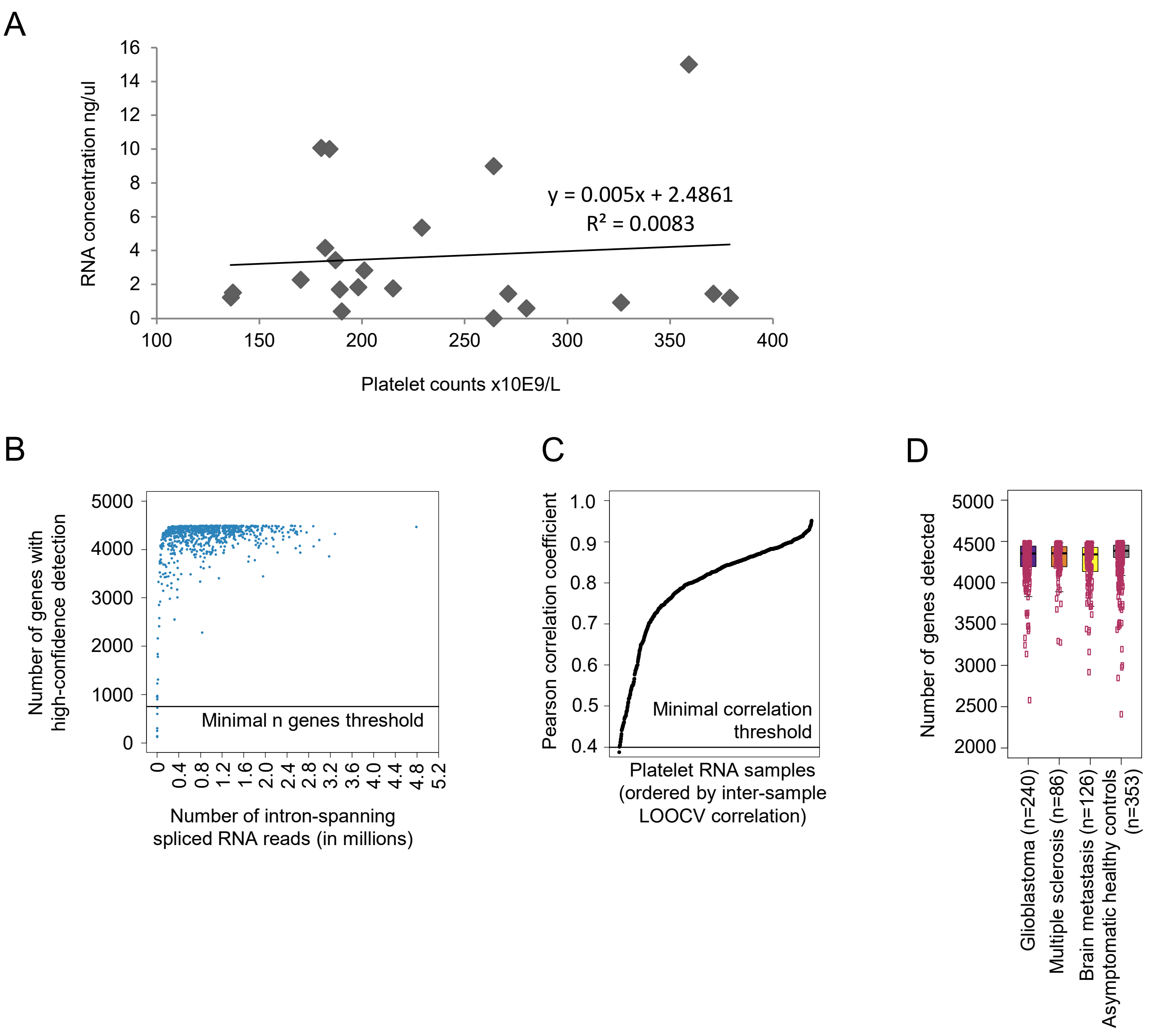

E

Training series

Predicted Class

Actual Class	Glio-blastoma	Brain meta	MS	RNAs: 341 Total (*n*)
Glio-blastoma	19 76%	4 16%	2 8%	25 (100%)
Brain Meta	4 16%	15 60%	6 24%	25 (100%)
MS	0 0%	4 16%	21 84%	25 (100%)

Overall accuracy: 73%, p<0.001

Evaluation series

Predicted Class

Actual Class	Glio-blastoma	Brain meta	MS	RNAs: 341 Total (*n*)
Glio-blastoma	16 70%	5 22%	2 8%	23 (100%)
Brain Meta	4 17%	16 70%	3 13%	23 (100%)
MS	0 0%	2 9%	21 91%	23 (100%)

Overall accuracy: 77%, p<0.001

Validation series

Predicted Class

Actual Class	Glio-blastoma	Brain meta	MS	RNAs: 341 Total (*n*)
Glio-blastoma	31 76%	5 12%	5 12%	41 (100%)
Brain Meta	11 14%	59 76%	8 10%	78 (100%)
MS	4 11%	7 18%	27 71%	38 (100%)

Overall accuracy: 75%, p<0.001

Figure S1. Technical details of thromboSeq and multiclass analysis. Related to Figures 1 and 2 and STAR methods.

(**A**) Scatterplot of platelet counts and RNA concentration from 21 glioma patient samples all taken before surgery. (**B**) Sequencing depth versus number of RNAs detected. (**C**) Leave-one-sample out cross-correlations (see STAR Methods), assessing the intersample comparability and filtering out samples with low inter-sample correlation. (**D**) Boxplots indicating number of RNAs detected per clinical group. Individual samples are indicated as dots. (**E**) Multiclass analysis ‘Glioblastoma versus Brain metastasis versus Multiple sclerosis’-algorithm, indicating confusion matrices of the training, evaluation and validation series. Indicated are detection rates as compared to the actual classes in percentages.

Figure S2

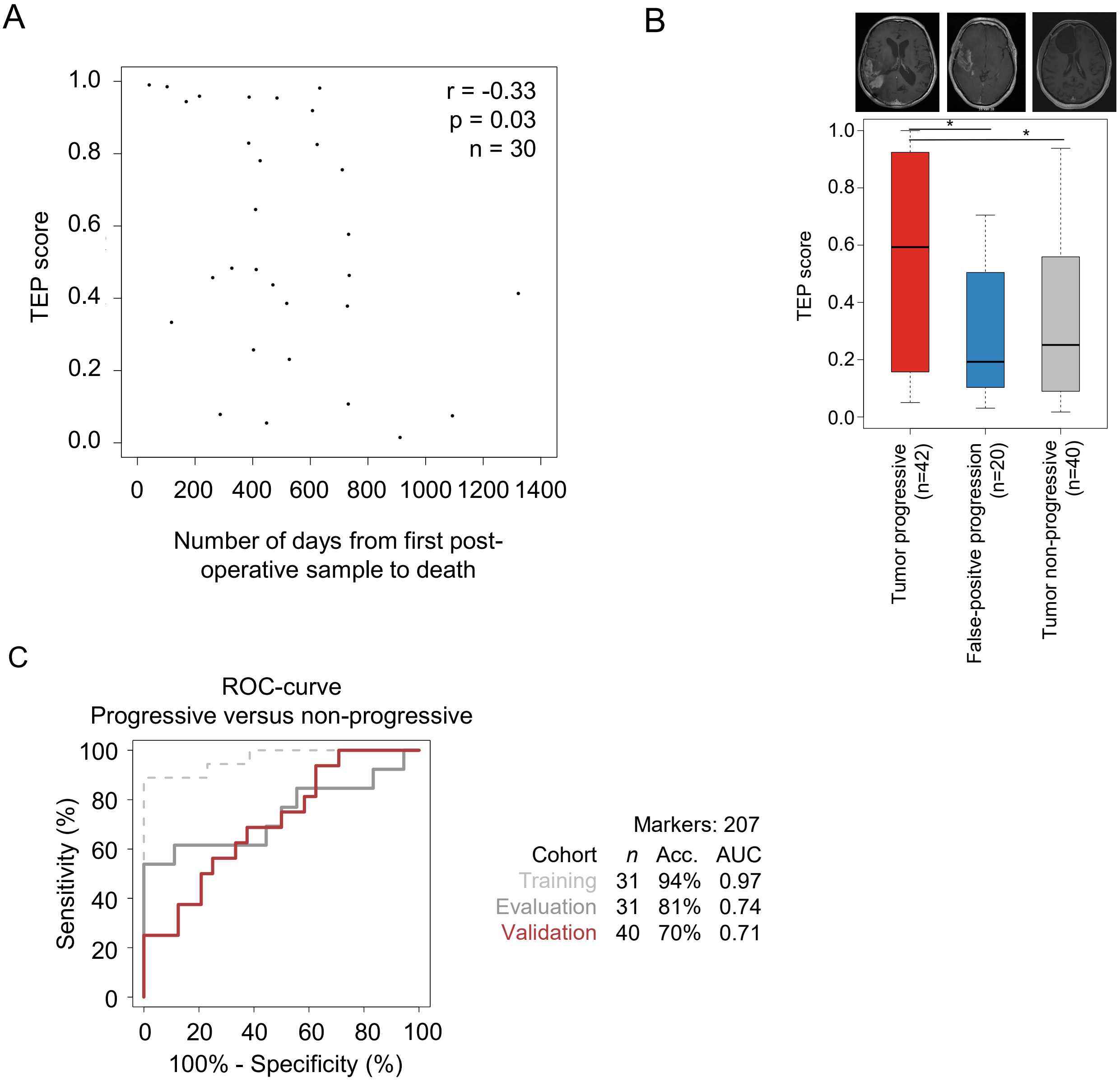
A
r = -0.33
p = 0.03
n = 30
TEP score
1.0
0.8
0.6
0.4
0.2
0.0
0
200
400
600
800
1000
1200
1400
Number of days from first post-operative sample to death
B
*
*
TEP score
1.0
0.8
0.6
0.4
0.2
0.0
Tumor progressive (n=42)
False-positve progression (n=20)
Tumor non-progressive (n=40)
C
ROC-curve
Progressive versus non-progressive
Sensitivity (%)
100
80
60
40
20
0
0
20
40
60
80
100
100% - Specificity (%)
Markers: 207
Cohort n Acc. AUC
Training 31 94% 0.97
Evaluation 31 81% 0.74
Validation 40 70% 0.71

Figure S2. Glioblastoma follow-up sample analysis. Related to Figure 2.

(**A**) Correlation plot of the post-operative TEP score and overall survival as determined from the day of first tumor resection. Each dot represents an individual patient. Indicated are the results of the Pearson's correlation analysis. (**B**) Boxplots of the TEP score from samples collected at moment of progressive tumor activity (n=42; red), false-positive progression (n=20; blue), and no progressive tumor activity (n=40; grey), as measured and confirmed by clinical observation and imaging modalities. The patients with false-positive progression show a reduced TEP score similar to those with no progressive tumor (Stable disease or regression), and as opposed to those with a progressive tumor. (**C**) ROC-curve of 'glioblastoma progression versus regression'-algorithm employing the conventional PSO-enhanced thromboSeq algorithm and the series as employed for digitalSWARM, indicating insufficient classification power employing the conventional PSO-enhanced thromboSeq algorithm. Included are the training series (dashed grey, n=31), evaluation series (grey, n=31), and validation series (red, in the digitalSWARM the separate verification and validation series, n=40). Indicated are sample series sizes, best accuracy, and AUC-value.

Figure S3

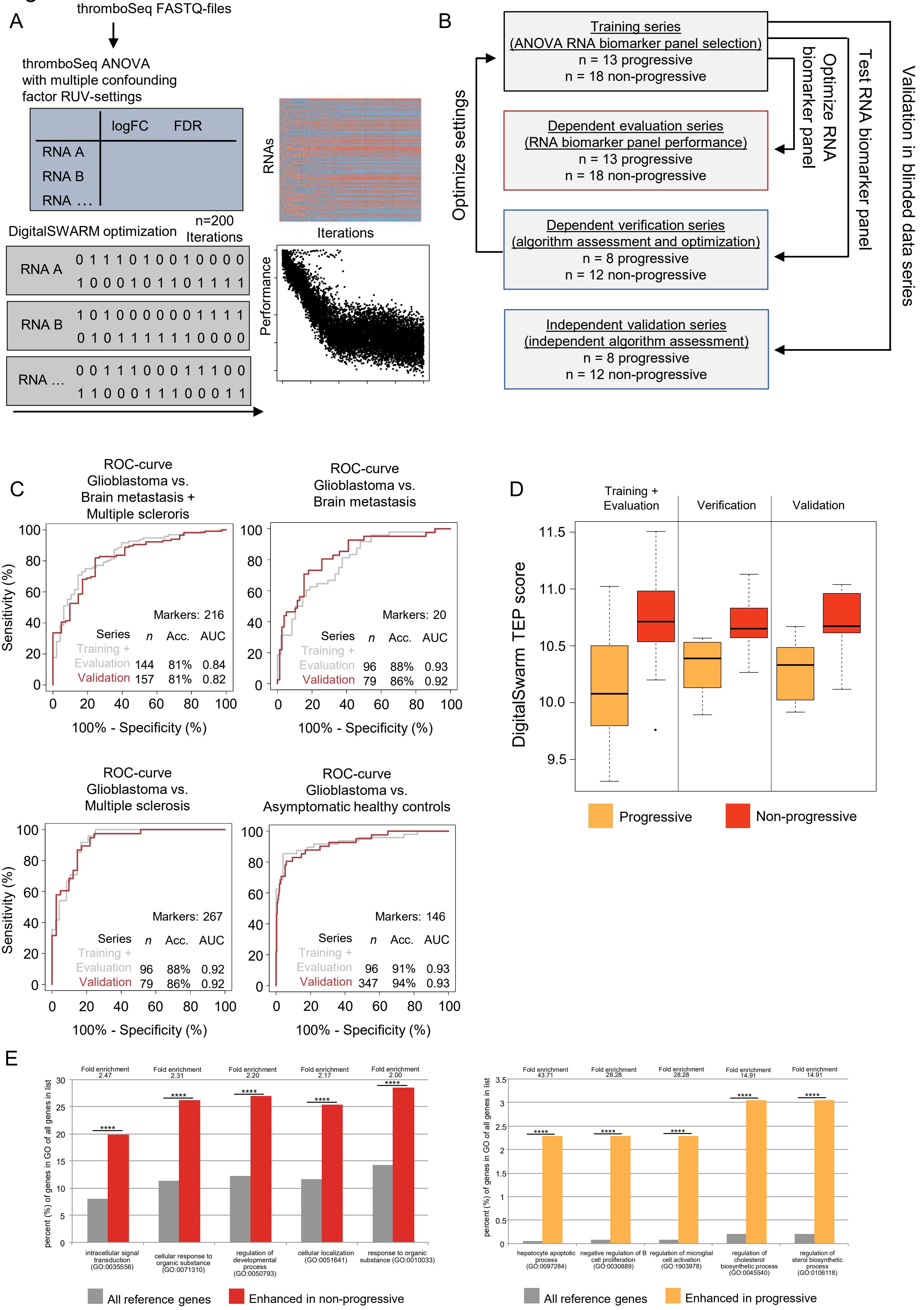

A
thromboSeq FASTQ-files
thromboSeq ANOVA with multiple confounding factor RUV-settings
logFC
FDR
RNA A
RNA B
RNA …
RNAs
Iterations
DigitalSWARM optimization
n=200 Iterations
RNA A
0 1 1 1 0 1 0 0 1 0 0 0 0
1 0 0 0 1 0 1 1 0 1 1 1 1
RNA B
1 0 1 0 0 0 0 0 0 1 1 1 1
0 1 0 1 1 1 1 1 1 0 0 0 0
RNA …
0 0 1 1 1 0 0 0 1 1 1 0 0
1 1 0 0 0 1 1 1 0 0 0 1 1
Performance
B
Training series
(ANOVA RNA biomarker panel selection)
n = 13 progressive
n = 18 non-progressive
Dependent evaluation series
(RNA biomarker panel performance)
n = 13 progressive
n = 18 non-progressive
Dependent verification series
(algorithm assessment and optimization)
n = 8 progressive
n = 12 non-progressive
Independent validation series
(independent algorithm assessment)
n = 8 progressive
n = 12 non-progressive
Optimize settings
Optimize RNA biomarker panel
Test RNA biomarker panel
Validation in blinded data series
C
ROC-curve Glioblastoma vs. Brain metastasis + Multiple scleroris
Sensitivity (%)
100% - Specificity (%)
Markers: 216
Series n Acc. AUC
Training + Evaluation 144 81% 0.84
Validation 157 81% 0.82
ROC-curve Glioblastoma vs. Brain metastasis
Markers: 20
Series n Acc. AUC
Training + Evaluation 96 88% 0.93
Validation 79 86% 0.92
ROC-curve Glioblastoma vs. Multiple sclerosis
Markers: 267
Series n Acc. AUC
Training + Evaluation 96 88% 0.92
Validation 79 86% 0.92
ROC-curve Glioblastoma vs. Asymptomatic healthy controls
Markers: 146
Series n Acc. AUC
Training + Evaluation 96 91% 0.93
Validation 347 94% 0.93
D
Training + Evaluation
Verification
Validation
DigitalSwarm TEP score
Progressive
Non-progressive
E
Fold enrichment 2.47
Fold enrichment 2.31
Fold enrichment 2.20
Fold enrichment 2.17
Fold enrichment 2.00
percent (%) of genes in GO of all genes in list
intracellular signal transduction (GO:0035556)
cellular response to organic substance (GO:0071310)
regulation of developmental process (GO:0050793)
cellular localization (GO:0051641)
response to organic substance (GO:0010033)
All reference genes
Enhanced in non-progressive
Fold enrichment 43.71
Fold enrichment 28.28
Fold enrichment 28.28
Fold enrichment 14.91
Fold enrichment 14.91
hepatocyte apoptotic process (GO:0097284)
negative regulation of B cell proliferation (GO:0030889)
regulation of microglial cell activation (GO:1903978)
regulation of cholesterol biosynthetic process (GO:0045540)
regulation of sterol biosynthetic process (GO:0106118)
All reference genes
Enhanced in progressive

Figure S3. Glioblastoma digitalSWARM analysis. Related to Figure 2.

(**A**) Schematic representation of digitalSWARM. Input thromboSeq FASTQ files are analyzed and summarized according to the described protocol. The best RUV-correction threshold is selected by grid analysis and multiple ANOVA-comparisons, ultimately selecting the ANOVA analysis with which the lowest FDR can be reached. These settings serve as input dataset for the iteratively optimizing digitalSWARM module, in- or excluding RNAs based on a binary inclusion decision step. With each iteration, the biomarker RNA panel becomes more stable, and the performance improves. (**B**) Schematic representation of the series employed for digitalSWARM; the training and evaluation series are employed for biomarker RNA panel selection and optimization. The verification series is employed for selection of the best training-evaluation series-combination. Following these optimization-steps the validation series is classified. (**C**) ROC-curves of the glioblastoma diagnostics datasets as presented in Figure 1B-E. Series were re-analyzed according to the digitalSWARM algorithm. Included are the combined training and evaluation series (grey) and validation series (red). Indicated are sample series sizes, best accuracy, and AUC-value. (**D**) Boxplots of the combinatorial TEP score calculated by digitalSWARM for progressive (yellow) and non-progressive (red) patients with glioblastoma for both the training, the evaluation, the verification, as the validation series. (**E**) Barplots of PANTHER gene ontology analyses indicating the fold enrichment of RNAs with enhanced spliced read junctions in the 'non-progressive'-group (left plot, red) and RNAs with enhanced spliced read junctions in the 'progressive'-group (right plot, yellow) as compared to all reference genes (grey). Indicated are fold enrichment scores and gene-ontology processes. **** = $p<0.0001$.

Table S1. Patient characteristics. Related to figure 1 and 2.

Detailed overview of all samples included in this study (n=851), including patient characteristics, and inclusion in the multiple algorithms developed in this study. Indicated are sample names, patient group, classification group, hospital of blood collection, storage time between blood collection and platelet isolation (blood storage time), age in years, gender, smoking, metastasis, treatment, and response to treatment. In addition, for each sample is stated to which cohort (training, evaluation or validation) the sample is assigned in the different classifiers. In column 'Hospital'; VUMC = VU University Medical Center, Amsterdam, The Netherlands, NKI = Dutch Cancer Institute, Amsterdam, The Netherlands, AMC = Academical Medical Center, Amsterdam, The Netherlands, UMCU = Utrecht Medical Center, Utrecht, The Netherlands, MGH = Massachusetts General Hospital, Boston, USA, VIENNA = the Medical University of Vienna, Austria, RAD = the Radboud University Medical Center Nijmegen, The Netherlands, MAAS = the University Hospital of Maastricht, The Netherlands. In column 'Gender'; M = male, F = female. In column 'Smoking' Y = current, N = never, F = former. In column 'Metastasis'; Y = yes, N = no. NA = not applicable.

Table S2A-F. Biomarker RNA panels. Related to figure 1 and 2

Biomarker RNA panels of the classification algorithms. Indicated for each biomarker panel are included the Ensembl IDs, HGNC symbols, and gene descriptions.

Data S1. Glioblastoma follow-up sample analysis. Related to Figure 2

TEP score plotted during the therapy course indicated as days since first tumor resection for 52 unique glioblastoma patients. The MR-images acquired around each time point are shown on top of the graph. Evaluation of tumor growth is indicated below each MR-image.

Data S1

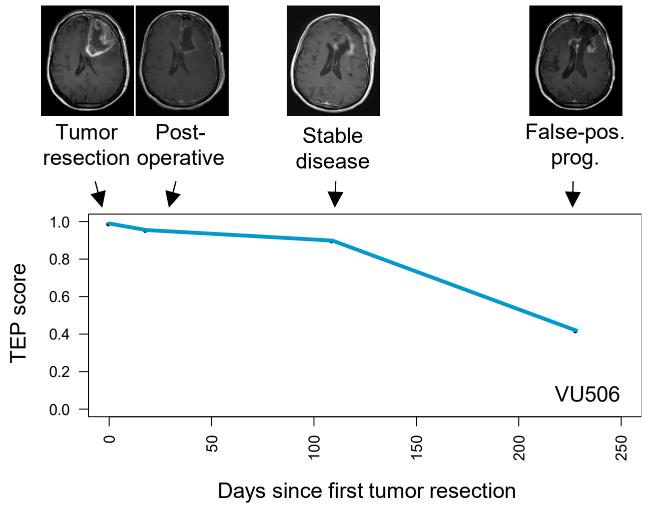
Tumor resection
Post-operative
Stable disease
False-pos. prog.
TEP score
1.0
0.8
0.6
0.4
0.2
0.0
VU506
0
50
100
150
200
250
Days since first tumor resection

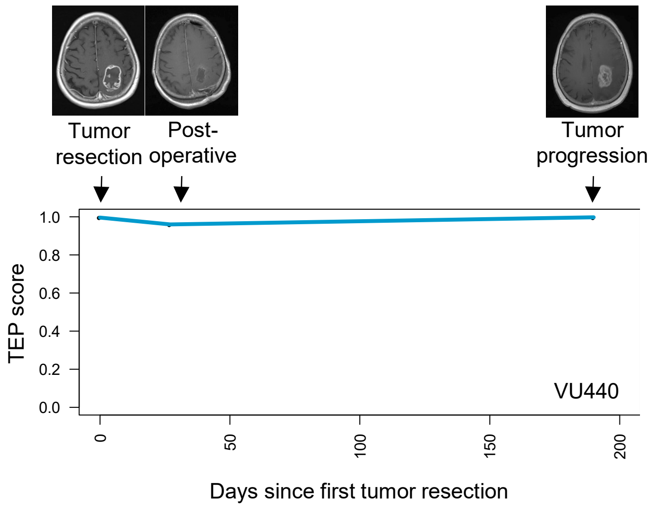
Tumor resection
Post-operative
Tumor progression
TEP score
1.0
0.8
0.6
0.4
0.2
0.0
VU440
0
50
100
150
200
Days since first tumor resection

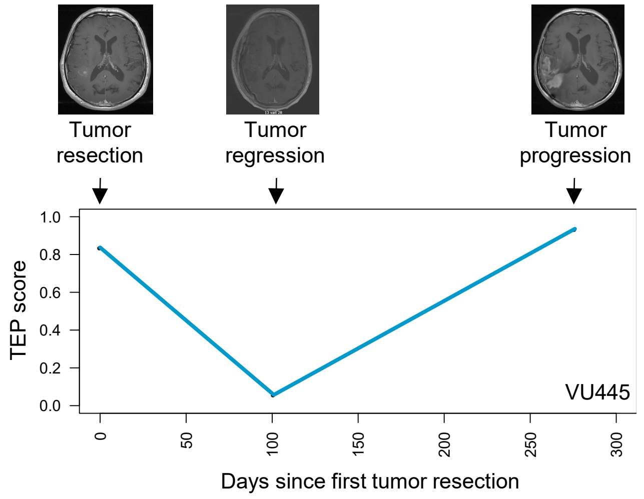
Tumor resection
Tumor regression
Tumor progression
TEP score
1.0
0.8
0.6
0.4
0.2
0.0
VU445
0
50
100
150
200
250
300
Days since first tumor resection

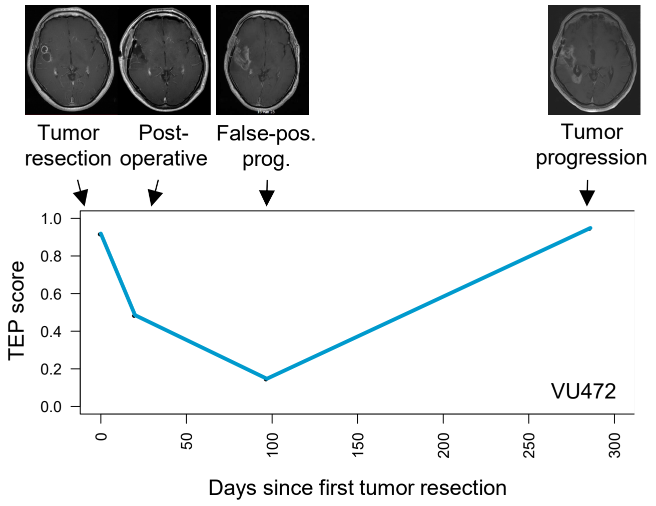
Tumor resection
Post-operative
False-pos. prog.
Tumor progression
TEP score
1.0
0.8
0.6
0.4
0.2
0.0
VU472
0
50
100
150
200
250
300
Days since first tumor resection

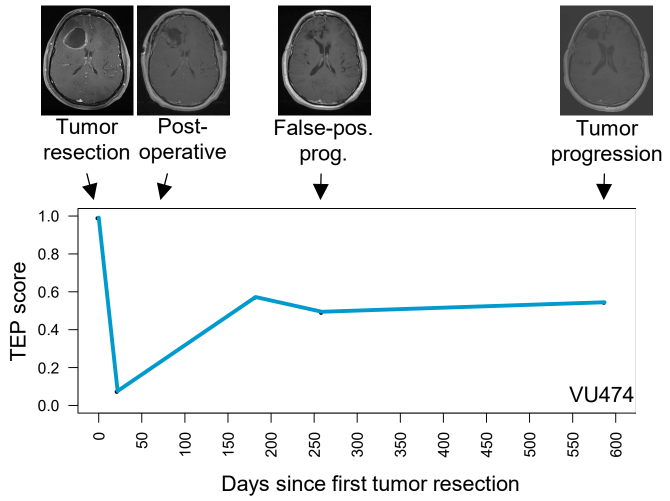
Tumor resection
Post-operative
False-pos. prog.
Tumor progression
TEP score
1.0
0.8
0.6
0.4
0.2
0.0
VU474
0
50
100
150
200
250
300
350
400
450
500
550
600
Days since first tumor resection

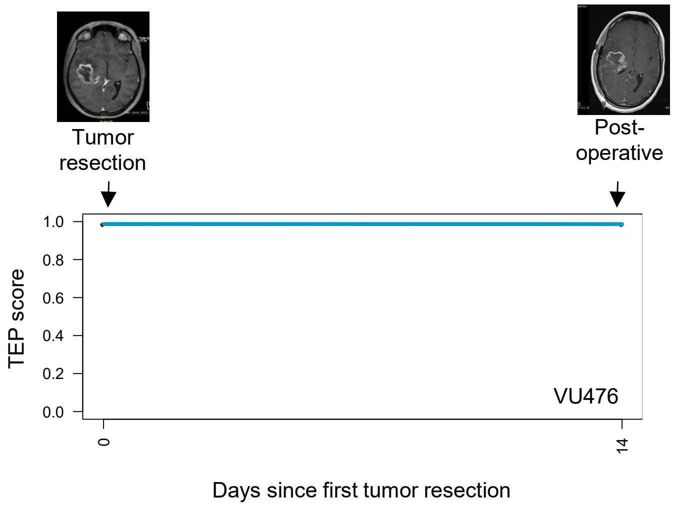
Tumor resection
Post-operative
TEP score
1.0
0.8
0.6
0.4
0.2
0.0
VU476
0
14
Days since first tumor resection

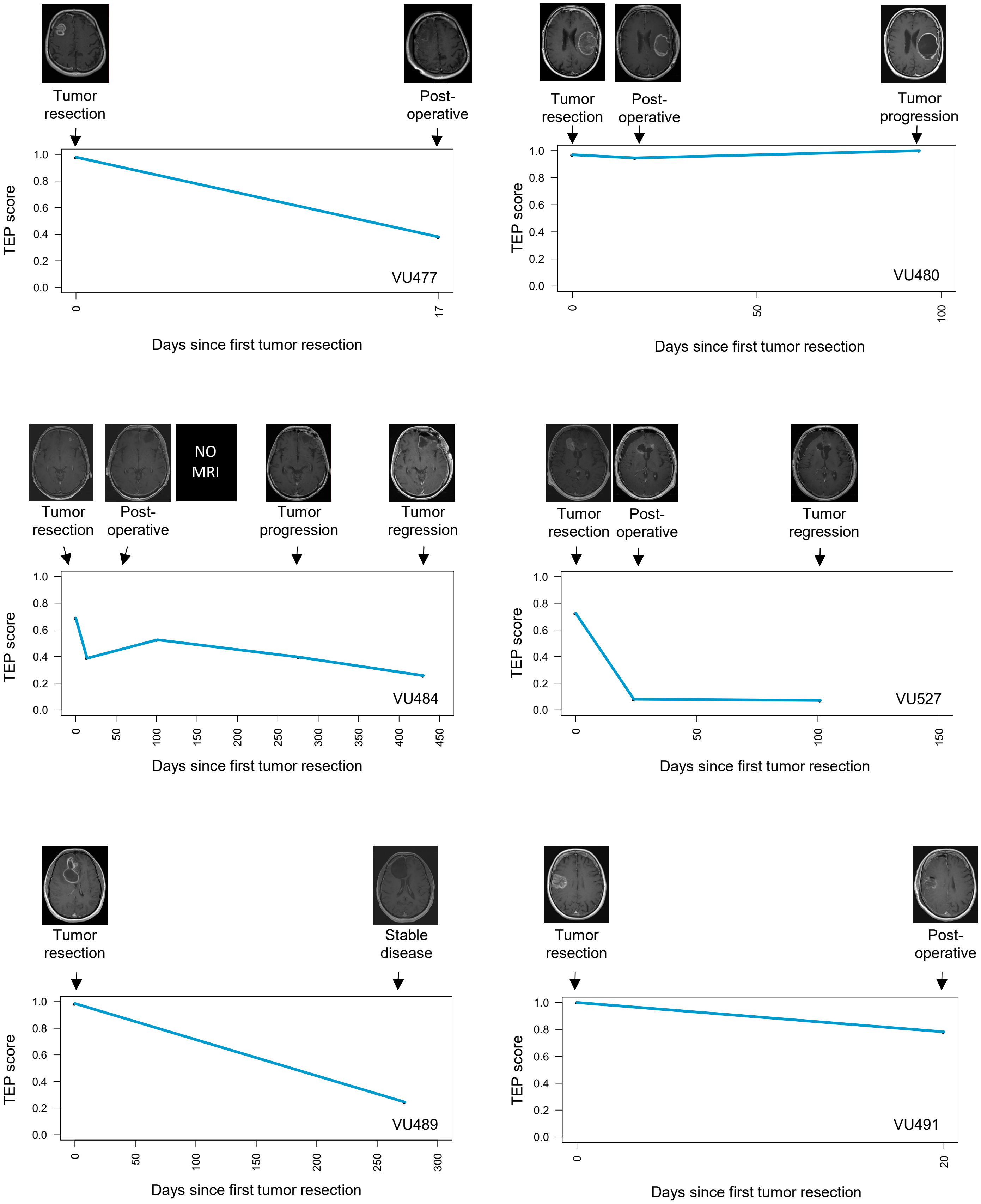
Tumor resection
Post-operative
TEP score
1.0
0.8
0.6
0.4
0.2
0.0
0
17
VU477
Days since first tumor resection
Tumor resection
Post-operative
Tumor progression
TEP score
0
50
100
VU480
Days since first tumor resection
NO MRI
Tumor resection
Post-operative
Tumor progression
Tumor regression
TEP score
0
50
100
150
200
250
300
350
400
450
VU484
Days since first tumor resection
Tumor resection
Post-operative
Tumor regression
TEP score
0
50
100
150
VU527
Days since first tumor resection
Tumor resection
Stable disease
TEP score
0
50
100
150
200
250
300
VU489
Days since first tumor resection
Tumor resection
Post-operative
TEP score
0
20
VU491
Days since first tumor resection

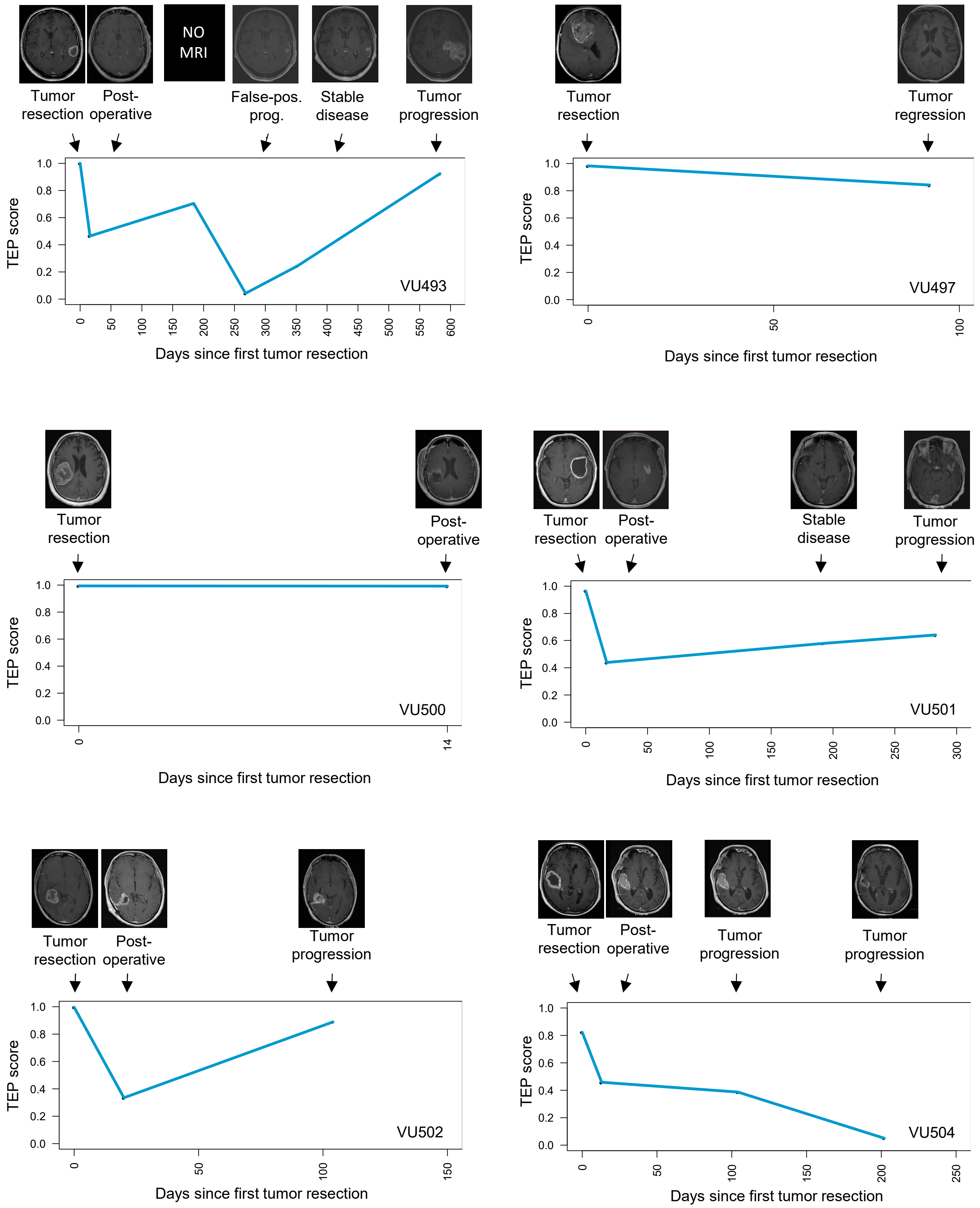
NO MRI
Tumor resection
Post-operative
False-pos. prog.
Stable disease
Tumor progression
TEP score
VU493
Days since first tumor resection
Tumor resection
Tumor regression
TEP score
VU497
Days since first tumor resection
Tumor resection
Post-operative
TEP score
VU500
Days since first tumor resection
Tumor resection
Post-operative
Stable disease
Tumor progression
TEP score
VU501
Days since first tumor resection
Tumor resection
Post-operative
Tumor progression
TEP score
VU502
Days since first tumor resection
Tumor resection
Post-operative
Tumor progression
Tumor progression
TEP score
VU504
Days since first tumor resection

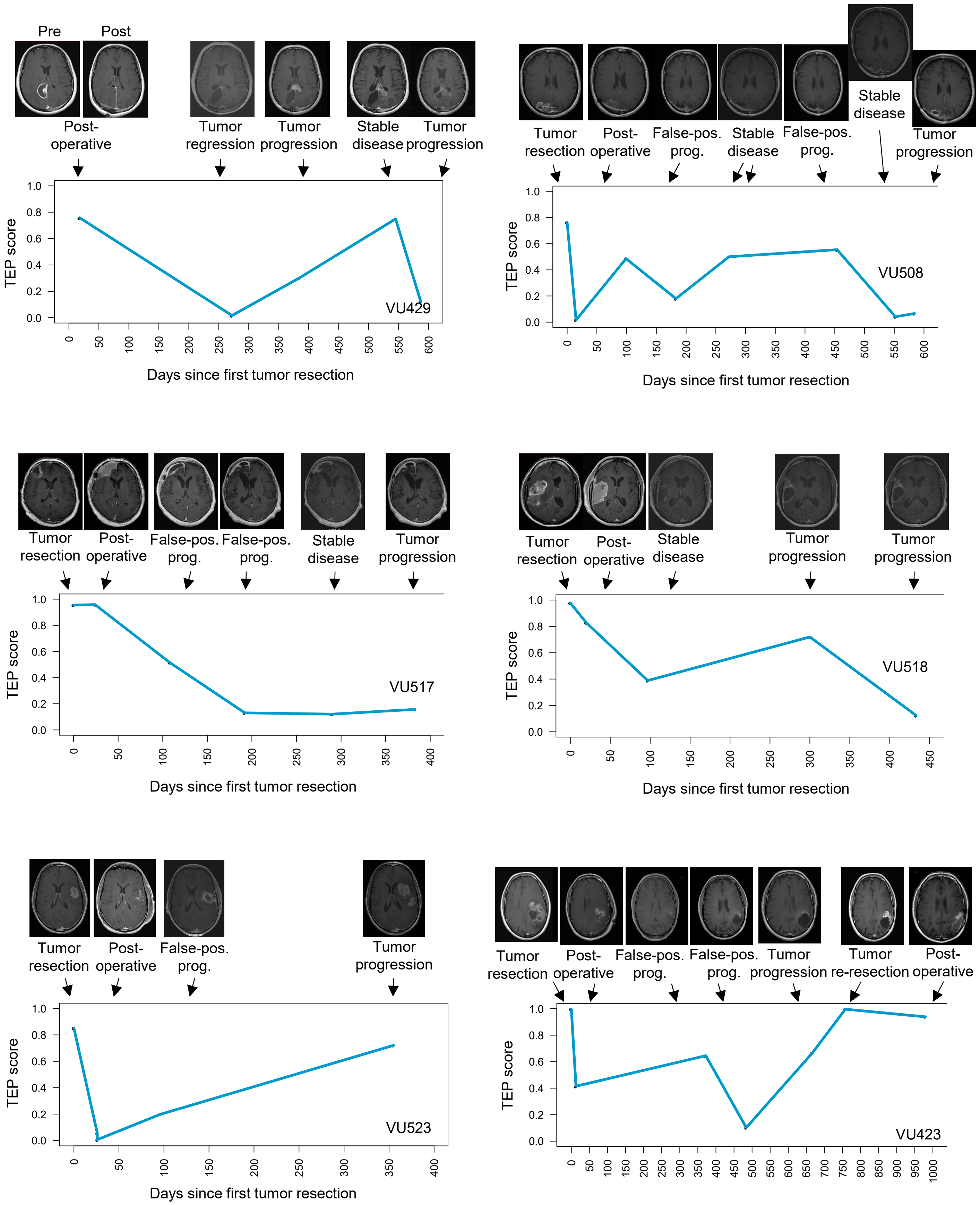
Pre
Post
Post-operative
Tumor regression
Tumor progression
Stable disease
Tumor progression
TEP score
Days since first tumor resection
VU429
Tumor resection
Post-operative
False-pos. prog.
Stable disease
False-pos. prog.
Stable disease
Tumor progression
TEP score
Days since first tumor resection
VU508
Tumor resection
Post-operative
False-pos. prog.
False-pos. prog.
Stable disease
Tumor progression
TEP score
Days since first tumor resection
VU517
Tumor resection
Post-operative
Stable disease
Tumor progression
Tumor progression
TEP score
Days since first tumor resection
VU518
Tumor resection
Post-operative
False-pos. prog.
Tumor progression
TEP score
Days since first tumor resection
VU523
Tumor resection
Post-operative
False-pos. prog.
False-pos. prog.
Tumor progression
Tumor re-resection
Post-operative
TEP score
VU423

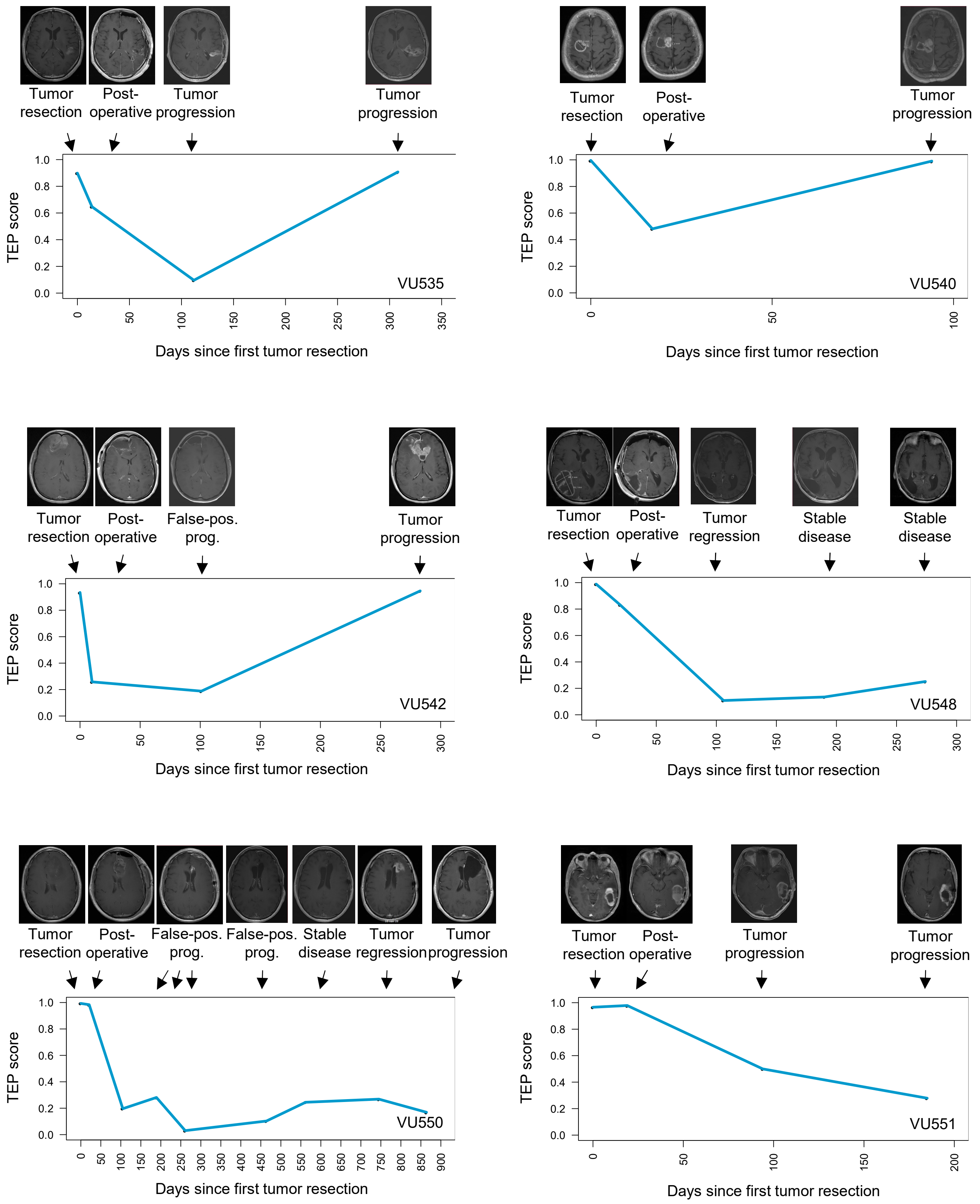
Tumor resection
Post-operative
Tumor progression
Tumor progression
TEP score
VU535
Days since first tumor resection
Tumor resection
Post-operative
Tumor progression
TEP score
VU540
Days since first tumor resection
Tumor resection
Post-operative
False-pos. prog.
Tumor progression
TEP score
VU542
Days since first tumor resection
Tumor resection
Post-operative
Tumor regression
Stable disease
Stable disease
TEP score
VU548
Days since first tumor resection
Tumor resection
Post-operative
False-pos. prog.
False-pos. prog.
Stable disease
Tumor regression
Tumor progression
TEP score
VU550
Days since first tumor resection
Tumor resection
Post-operative
Tumor progression
Tumor progression
TEP score
VU551
Days since first tumor resection

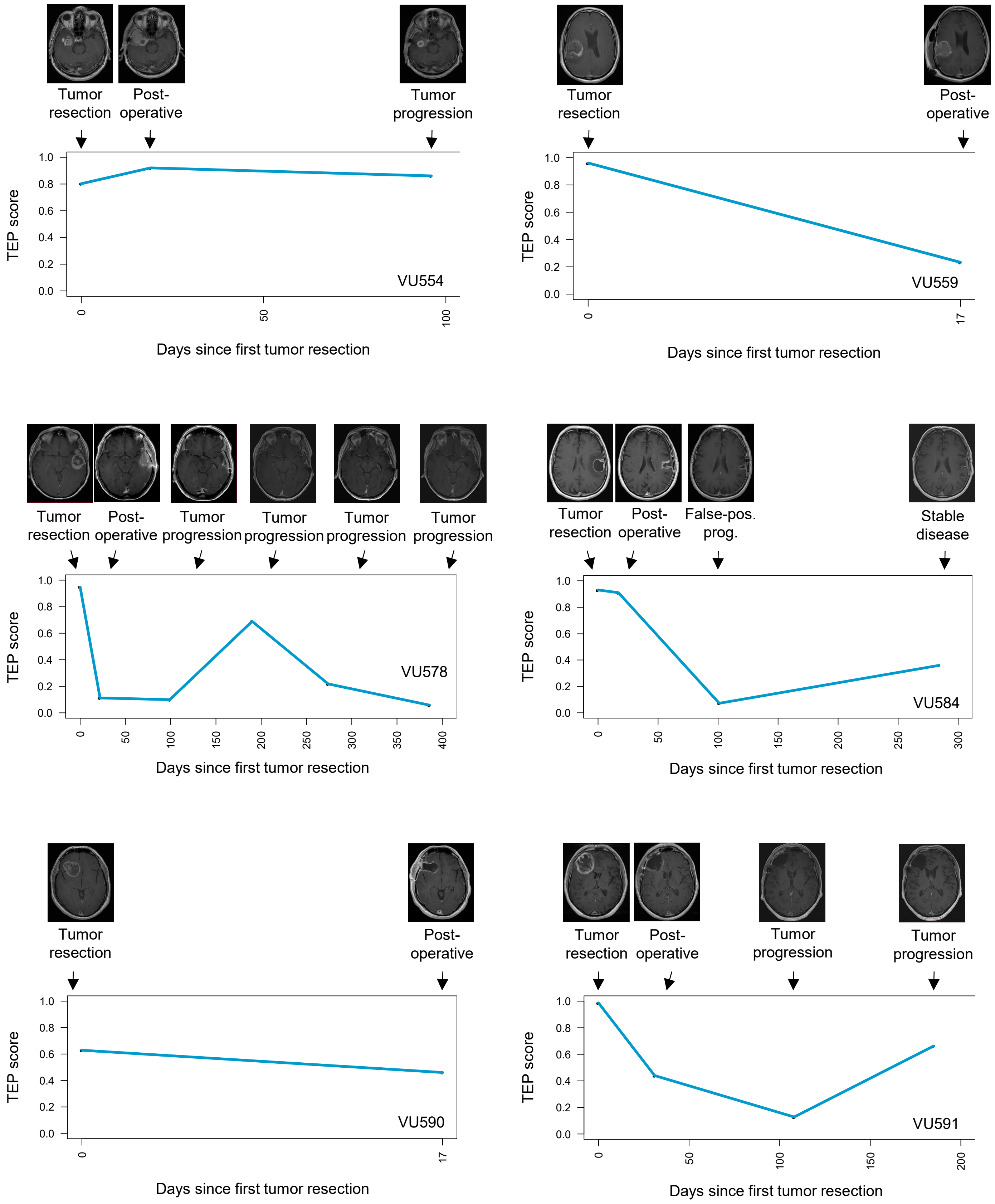

Tumor resection
Post-operative
Tumor progression
TEP score
1.0
0.8
0.6
0.4
0.2
0.0
0
50
100
VU554
Days since first tumor resection
Tumor resection
Post-operative
TEP score
1.0
0.8
0.6
0.4
0.2
0.0
0
17
VU559
Days since first tumor resection
Tumor resection
Post-operative
Tumor progression
Tumor progression
Tumor progression
Tumor progression
TEP score
1.0
0.8
0.6
0.4
0.2
0.0
0
50
100
150
200
250
300
350
400
VU578
Days since first tumor resection
Tumor resection
Post-operative
False-pos. prog.
Stable disease
TEP score
1.0
0.8
0.6
0.4
0.2
0.0
0
50
100
150
200
250
300
VU584
Days since first tumor resection
Tumor resection
Post-operative
TEP score
1.0
0.8
0.6
0.4
0.2
0.0
0
17
VU590
Days since first tumor resection
Tumor resection
Post-operative
Tumor progression
Tumor progression
TEP score
1.0
0.8
0.6
0.4
0.2
0.0
0
50
100
150
200
VU591
Days since first tumor resection

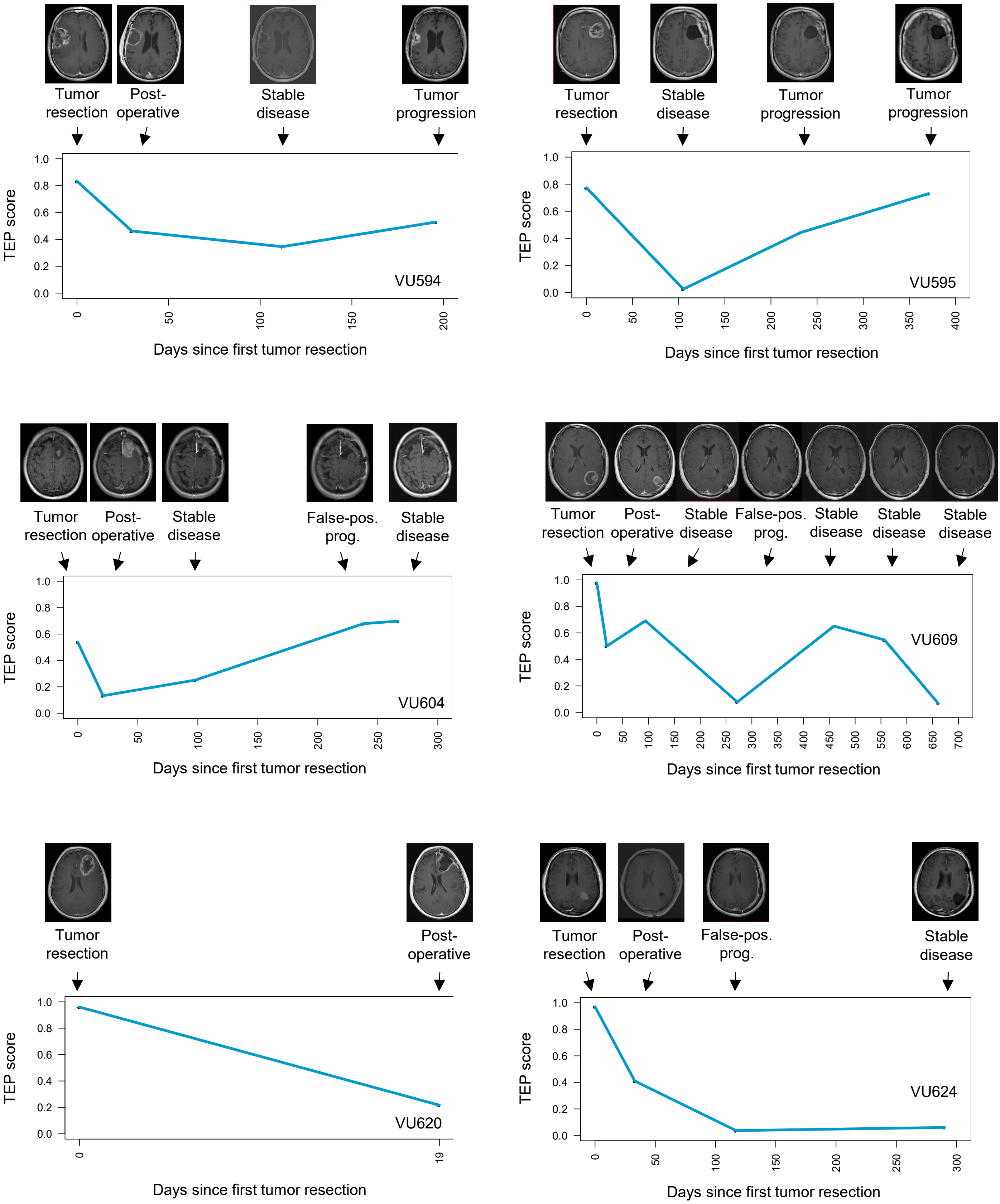

Tumor resection
Post-operative
Stable disease
Tumor progression
TEP score
VU594
Days since first tumor resection
Tumor resection
Stable disease
Tumor progression
Tumor progression
TEP score
VU595
Days since first tumor resection
Tumor resection
Post-operative
Stable disease
False-pos. prog.
Stable disease
TEP score
VU604
Days since first tumor resection
Tumor resection
Post-operative
Stable disease
False-pos. prog.
Stable disease
Stable disease
Stable disease
TEP score
VU609
Days since first tumor resection
Tumor resection
Post-operative
TEP score
VU620
Days since first tumor resection
Tumor resection
Post-operative
False-pos. prog.
Stable disease
TEP score
VU624
Days since first tumor resection

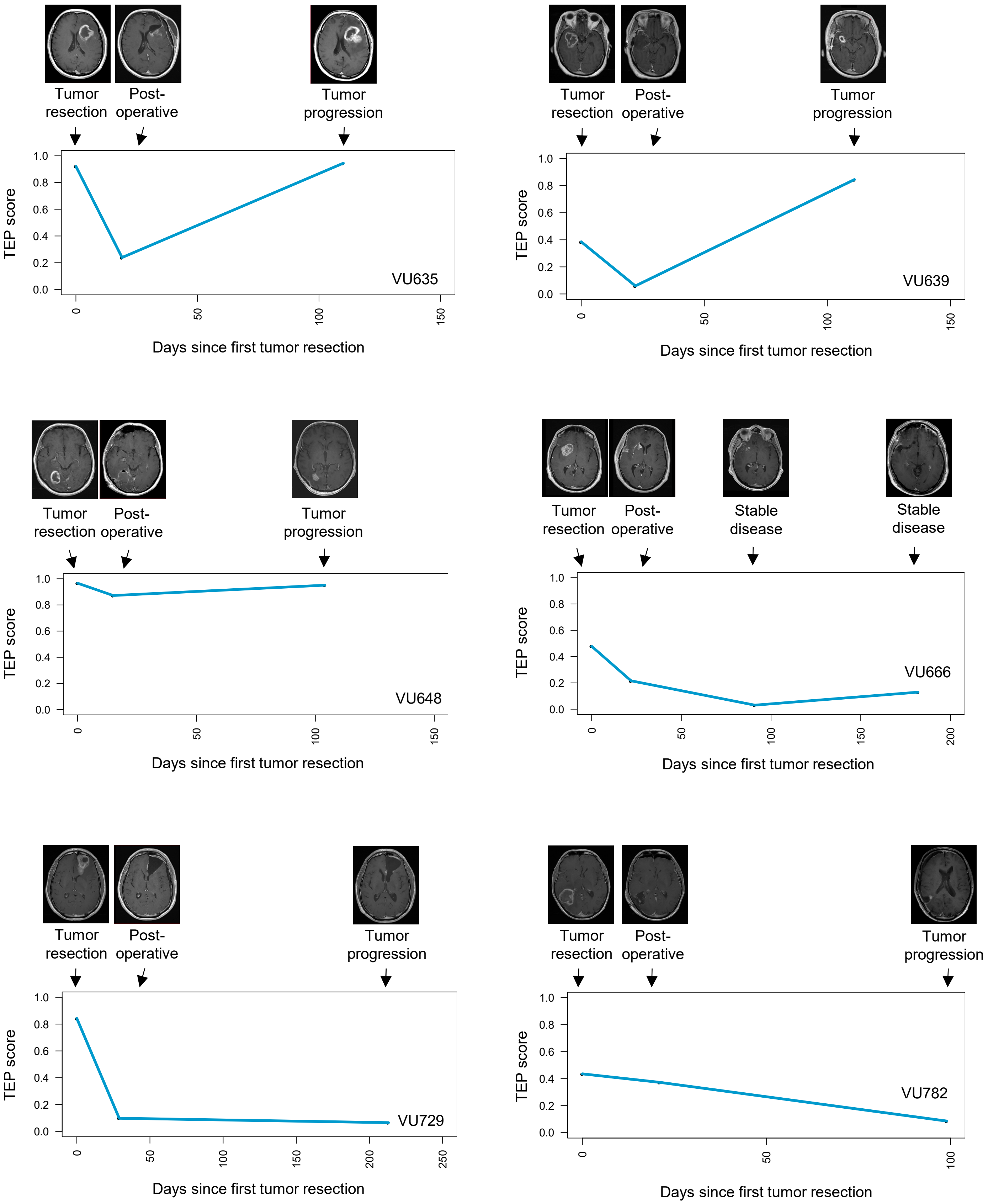
Tumor resection
Post-operative
Tumor progression
TEP score
1.0
0.8
0.6
0.4
0.2
0.0
0
50
100
150
Days since first tumor resection
VU635
Tumor resection
Post-operative
Tumor progression
TEP score
VU639
Days since first tumor resection
Tumor resection
Post-operative
Tumor progression
TEP score
VU648
Days since first tumor resection
Tumor resection
Post-operative
Stable disease
Stable disease
TEP score
200
VU666
Days since first tumor resection
Tumor resection
Post-operative
Tumor progression
TEP score
250
VU729
Days since first tumor resection
Tumor resection
Post-operative
Tumor progression
TEP score
VU782
Days since first tumor resection

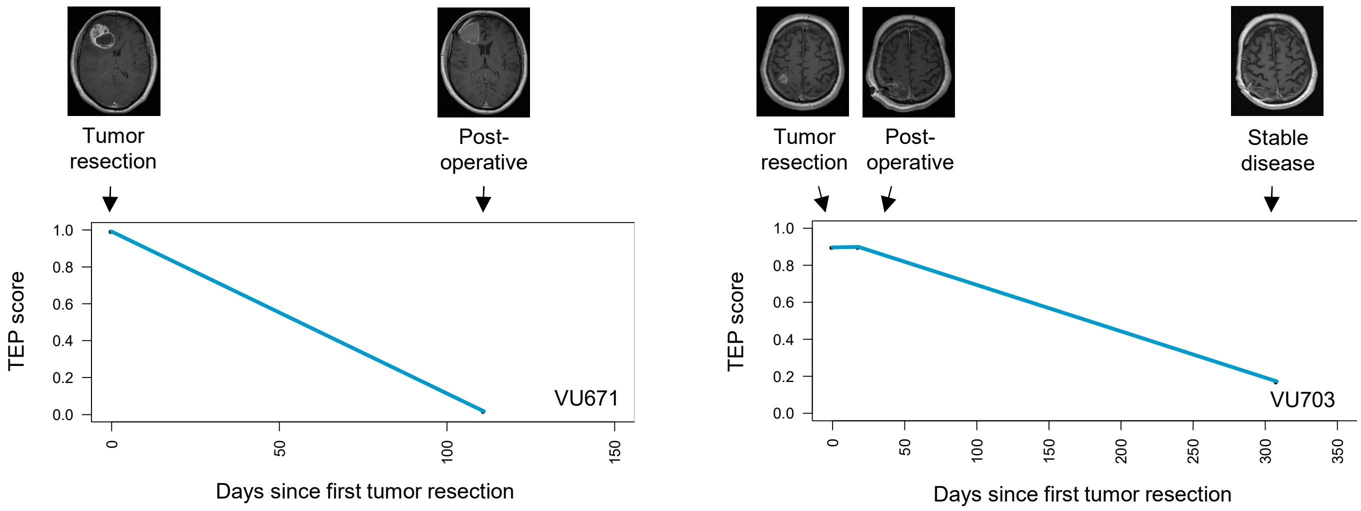

Data S1. Glioblastoma follow-up sample analysis. Related to Figure 2

TEP score plotted during the therapy course indicated as days since first tumor resection for 52 unique glioblastoma patients. The MR-images acquired around each time point are shown on top of the graph. Evaluation of tumor growth is indicated below each MR-image.
